# Concentration Variability of Water-Soluble Ions during the Acceptable and Exceeded Pollution in an Industrial Region

**DOI:** 10.3390/ijerph17103447

**Published:** 2020-05-15

**Authors:** Barbora Švédová, Helena Raclavská, Marek Kucbel, Jana Růžičková, Konstantin Raclavský, Miroslav Koliba, Dagmar Juchelková

**Affiliations:** 1 Centre ENET—Energy Units for Utilization of Non-Traditional Energy Sources, VŠB—Technical University of Ostrava, 17. listopadu 15/2172, 708 00 Ostrava-Poruba, Czech Republic; barbora.svedova@vsb.cz (B.Š.); helena.raclavska@vsb.cz (H.R.); jana.ruzickova@vsb.cz (J.R.); konstantin.raclavsky@vsb.cz (K.R.); 2 Diabetic and podiatric clinic, Frýdecká 936/59, 739 32 Vratimov, Czech Republic; mudrkoliba@mudrkoliba.cz; 3 Department of Electronics, VŠB—Technical University of Ostrava, Faculty of Electrical Engineering and Computer Science, 17. listopadu 15/2172, 708 00 Ostrava-Poruba, Czech Republic; dagmar.juchelkova@vsb.cz

**Keywords:** water-soluble inorganic ions, particulate matter, meteorological parameters, enrichment factor, air pollution

## Abstract

This study investigates the chemical composition of water-soluble inorganic ions at eight localities situated in the Moravian–Silesian Region (the Czech Republic) at the border with Poland. Water-soluble inorganic ions were monitored in the winter period of 2018 (January, 11 days and February, 5 days). The set was divided into two periods: the acceptable period (the 24-h concentration of PM_10_ < 50 µg/m^3^) and the period with exceeded pollution (PM_10_ ˃ 50 µg/m^3^). Air quality in the Moravian–Silesian Region and Upper Silesia is among the most polluted in Europe, especially in the winter season when the concentration of PM_10_ is repeatedly exceeded. The information on the occurrence and behaviour of water-soluble inorganic ions in the air during the smog episodes in Europe is insufficient. The concentrations of water-soluble ions (chlorides, sulphates, nitrates, ammonium ions, potassium) during the exceeded period are higher by two to three times compared with the acceptable period. The major anions for both acceptable period and exceeded pollution are nitrates. During the period of exceeded pollution, percentages of water-soluble ions in PM_10_ decrease while percentages of carbonaceous matter and insoluble particles (fly ash) increase.

## 1. Introduction

The particle pollution (particulate matter—PM) represents one of the critical parameters for monitoring of ambient air quality. The effect of particulate matter on health is summarized in review [1]. Depending on the parameters of atmospheric pollution, the characteristics of the persons exposed and exposure, various forms of the disease may develop, e.g., a local or systemic disease, a curable or incurable disease, a state of simple disease, or pathological conditions complicated by persistent long-term effects. The association between exposure to polluted air and diseases of the cardiovascular system (atherosclerosis, coronary heart disease) and diseases of the respiratory system (bronchitis, pneumoconiosis, inhalation pneumonia, some types of respiratory cancer, bronchial asthma) is already explored in great detail [2]. Particulate matter contains insoluble particles: (fly ash) + resuspended particles (salts, pavement dust) + organic/elemental carbon, the water-soluble inorganic ions (WSSI), and metals present in both soluble and insoluble form.

Particulate matter was classified by the International Agency for Research on Cancer (IARC) as carcinogenic to humans (Group 1). Ambient air pollution and particularly airborne particulate matter cause adverse human health effects including respiratory illnesses, cardiovascular diseases: myocardial infarction, cardiac arrhythmias, ischemic stroke, vascular dysfunction, hypertension and atherosclerosis [3], carcinogenic effects, and is adverse to asthma, premature death, diabetes [4], and shortens life expectancy [5,6]. Children in the industrial Ostrava conurbation have the high incidence of acute illnesses and allergies compared with children from other regions of the Czech Republic [7]. 

Air pollution can also generate psychological impacts on individuals or groups, e.g., reduce subjective well-being, cause anxiety and depression, and even increase suicide risk [8]. The health effects of exposure to aerosol particle matter are well documented and therefore is necessary for the improvement of air quality to used reference guidelines for ambient particulate concentration: the World Health Organisation Air Quality Guidelines (PM_10_ 24-h mean: 50 µg/m^3^) [9]. At present, Directive 2008/50/EC of the European Parliament and of the Council on ambient air quality and cleaner air for Europe does not specify the value for determining the smog conditions based on concentrations of PM_10_ [10]. In the Czech Republic, the smog situation is declared under the Air Protection Act 201/2012 if at least one parameter exceeds the pollution levels: sulphur dioxide (250 μg.m^3^/hour), nitrogen dioxide (200 µg/m^3^/hour), PM_10_ (100 µg/m^3^/12 h), or tropospheric ozone (180 µg/m^3^/hour) [11]. Stable weather conditions, together with the extensive use of coal combustion, often lead to severe smog episodes in some urban environments, especially in Eastern Europe [12]. 

The smog measures in the Moravian-Silesian Region (MSR) were declared during 2010 for the total time of 1031 h. The smog measures during 2018 were declared for 336 h. The shortest time of the smog conditions was 124 h in 2015. The year 2015 was characterized by the above-average temperature in winter and better dispersion conditions compared with other years [13].

The air quality not only in the Czech Republic (specifically in Moravian–Silesian Region), but also in Europe has been improved significantly over the previous decades. Nevertheless, air pollution is still a large environmental health risk problem. The Ostrava-Karviná conurbation representing part of the Ostrava Basin is considered one of the most polluted areas in the European Union due to high concentrations of the most significant air pollutants, predominately airborne dust [14] and higher concentration of benzo(a)pyrene [15].

The study area is formed by communities and the City of Ostrava located in Moravian-Silesian Region (MSR) in the Czech Republic. The study area is located at the geomorphological unit of Ostrava Basin, which is part of the geomorphological region Western Outer Carpathian Depression. It is situated at the boundary of Northern Moravia, Silesia and Southern Poland. The region can be characterised as industrial with important energy production, metallurgy, coal mines, power engineering, chemical industry and others. The most important sources of pollution are energy production and metallurgy (production of coke, pig iron and steel). Air mass flowing represents an essential factor which influences the origin of smog episodes. The prevailing directions of air flowing are southwest and northeast. A significant decrease in pollutant dispersion in the atmosphere is caused by a decrease in the air flowing speed below 1.5 m/s. The calm periods represent up to 25% of the year. The territory of the City Ostrava has above-average occurrences of calm weather. The weather conditions combined with high industrialization of regions in the Czech Republic and Poland influence the origin of long smog episodes with concentrations of PM_10_ in the atmosphere at the level of hundreds of micrograms [16] Air pollution is concentrated mainly during poor dispersion conditions, especially in the winter season [17]. 

The same situation was observed in the border with Poland, especially in the Upper Silesian region and in Małopolska regions. These regions located in the southern part of Poland (at the border with the Czech Republic) are one of the most polluted regions in Europe [18]. Poor air quality is caused by an excessive concentration of PM_10_ [19,20]. The winter episodes with the high concentrations of PM_10_ in the southern cities of Poland (Zabrze, Krakow) are more influenced by local pollution than long-range transport and regional transport particles [20]. The primary pollution sources of PM_10_ in the area of Polish and Czech border between Ostrava and Katowice (Silesia Region) is residential heating [21]. During the years 2006–2012, the PM_10_ trans-boundary transport was more significant from the direction of Poland to the Czech Republic [17]. Wielgosiński and Czerwińska [20] defined characteristics of “Polish smog”. The most important emission source of particulate matter in Poland are the so-called “low emissions” (height up to 40 m), i.e., residential boilers and traffic pollution. “Polish smog” is characterised by varied meteorological conditions and high concentrations of sulphate and ammonium ions. The main source of ammonia emissions is the combustion of biomass and coal [20]. The ammonia emission factor for domestic stove for anthracite is 0.20 g/kg, for bituminous coal from 0.7 to 1.52 g/kg, and for biomass 0.72–1.08 g/kg [22]. The main sources of NH_3_ in air aerosol are not yet unequivocally identified. A study of the isotopic composition shows that, in China, 38–52% of NH_3_ emissions come from burning fossil fuels [23]. Car transport is another reason for the increase NH_3_ in air aerosol. Trying to reduce NO_X_ emissions from diesel engines by using urea (NH_2_)_2_CO reduces NOx output but also increases NH_3_ emissions, causing problems for the future [24]. Emissions of NH_3_ in the atmosphere react with SO_2_ which significantly decreases its concentrations. Therefore, ammonium sulphate is the main constituent of PM in “Polish dusty smog” [20].

This study is focused on obtaining the information on changes in the chemical composition of water-soluble ions, trace elements and forms of carbon occurrence in relationships to the direction of air flowing and concentration of PM_10_ (acceptable and exceeded pollution) to identify a source of pollution.

## 2. Materials and Methods

### 2.1. Sampling

**Figure 1:**
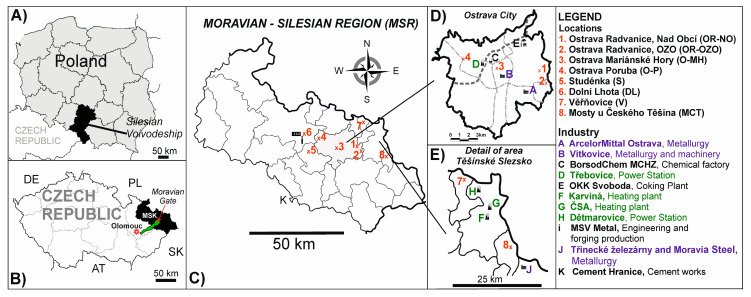
(**A**) Border between Czech Republic and Poland; (**B**) Localization of the Moravian-Silesian Region and Olomouc in the Czech Republic; (**C**) Detail of the Moravian-Silesian Region and localization of the sampling sites; The main pollution sources in Ostrava City (**D**) and in the area of Těšínské Slezsko (**E**).

Eight localities were selected where the influences of various emission sources were assumed (Supplementary Table S1 and Figure 1). In the text, we have used an abbreviation for these localities: Ostrava-Radvanice, Nad Obcí (OR-NO); Ostrava-Radvanice, OZO (OR-OZO); Ostrava-Mariánské Hory (O-MH); Ostrava-Poruba (O-P), Studénka (S), Dolní Lhota (DL), Věřňovice (V) and Mosty u Českého Těšína (MCT). For comparison, the locality Olomouc-Svatý Kopeček (Olomouc), was selected as the background locality, without industry emissions and high traffic influence. It is a rural locality near the Zoo of Olomouc (Supplementary Table S1 and Figure 1).

Identification and quantification of selected metals in Ostrava and Olomouc were performed by X-ray fluorescence spectrometry. The sampling of particles PM_10_ was performed on filters of 150 mm diameter with quartz fibres by high-volume samplers DHA 80 or MD05 produced by DIGITEL Co. with an airflow of 0.5 to 1.13 m^3^/min (Supplementary Table S1). Wind speed and direction were measured by the ultrasonic anemometer during 24 h in the winter season (18 January–28 January 2018) and during 12 h in the period of smog episode (9–13 February). The sampling of PM_10_ in Olomouc was performed as 24 h measurements from 21 November to 28 November 2018. Measurements in both areas were taken during heating seasons allowing comparison of measured data from different regions. The heating season in the Czech Republic begins on 1 September and ends on 31 May of the following year (Decree No 194/2007 Coll. of the Ministry of Industry and Trade, as amended) [25]. The measurements in Olomouc were made under conditions typical of the heating season. The division of days between ordinary winter days and days of smog episode was performed based on the announcement of smog situation for PM_10_ (100 µg/m^3^ per 12 h) according to the Ministry of the Environment (Act 201/2012 Coll.) [11]. The period from 18 January to 28 January 2018 is an example of typical winter conditions while the period from 9 February to 13 February represents a smog situation lasting for 78 h. Concentrations of PM_10_ decreased in the period from 11 to 13 February below 80 µg/m^3^. In total, 54 samples were obtained (in the periods of 12 h) during the smog episode. Average values of the results of chemical analyses were calculated in order to correspond to the sampling, which lasted 24 h. In total, 40 samples were obtained in the winter season. An original aim of the statistical evaluation of the results was the division of the whole set into the winter season and the smog episode. Even during the season from 22 to 23 January 2018, concentrations of PM_10_ reached values higher than 100 µg/m^3^ at two localities, and thus influenced the results of the chemical composition of PM_10_ in the winter season. Measured data in both sets for the winter season and smog episode were divided using the 24-h air concentration limit valid for PM_10_ (50 μg/m^3^) according to the Act No. 201/2012 Coll., on Air Pollution [11] and Directive 2008/50/EC of the European Parliament and of the Council of 21 May 2008 on ambient air quality and cleaner air for Europe [26]. Concentrations of PM_10_ over 50 μg/m^3^ are designated as the period of exceeded pollution, and period with the concentration of PM < 50 μg/m^3^ is designated as the period of acceptable pollution.

### 2.2. Analysis Method

One-quarter of each filter substrate was extracted with 25 mL deionized water in a PET vial for 24 h. After passing through microporous membranes with a 0.22 µm pore size, the extracts were analysed by ion chromatography. Determinations of water-soluble ions SO_4_^2−^, NO_3_^−^, NH_4_^+^, Cl^−^, Na^+^, K^+^, Ca^2+^, Mg^2+^, PO_4_^3−^, pH value, and conductivity in the particulate matter were performed by analysis of water extract by ion chromatography with the instrument 850 Professional IC (Metrohm AG, Herisau, Switzerland) according to the “Standard ISO 14911 Water quality: Determination of dissolved Li^+^, Na^+^, NH_4_^+^, K^+^, Mn^2+^, Ca^2+^, Mg^2+^, Sr^2+^, and Ba^2+^ using ion chromatography” and “Standard EN ISO 10304 Water quality: Determination of dissolved anions by liquid chromatography of ions”. Water-soluble organic carbon (WSOC) was determined according to the methodology of Karthikeyan and Balasubramanian [27] using ion chromatography (IC). Mineralisation of atmospheric aerosol particles, as well as further analyses of trace elements, were performed by inductively coupled plasma-optical emission spectrometry [28]. Determinations were performed for 18 elements: Al, As, Be, Ca, Cd, Co, Cu, Fe, K, Mn, Mo, Ni, Pb, Sb, Ti, Tl, V, and Zn (italics denote elements with concentrations below the detection limit). The concentrations of Ca and K after total decomposition (total content) were reduced by subtraction of the concentration of water-soluble ions and consequently used to calculate MD according to the Equation (1). 

The mineralogical phase analysis of particles was performed by X-ray diffraction (Diffractometer Bruker Advance D8, Bruker Corporation, Billerica, MA, USA) and used for verification of the presence of water-insoluble mineral phases (gypsum, zincite). The determinations of organic carbon (OC) and elemental carbon (EC) were performed using filters by thermo-optical analysis at the OC/EC Analyser (Sunset Laboratory Inc., Portland-Tigard, OR, USA). Organic matter (OM) was determined as OC multiplied by a factor of 1.4, which is commonly used for both urban areas as used e.g., in ref. [29] and rural areas [30]. The concentrations of OC/EC were measured using the method of the temperature programme EUSAAR 2 with the modification of thermo-optical transmittance. The statistical analysis and the correlation analysis (Spearman correlation coefficient) at the 0.05 level of significance were performed using the statistical software OriginPro 8.5 (OriginLab Corporation, Northampton, MA, USA).

## 3. Results and Discussion

In 2018, the acceptable daily limit for air concentration of PM_10_ (50 µg/m^3^) was most often exceeded at the locality V (94 times). Exceeding the air concentration limit was also very important at other localities: OR-NO (89×), OR-OZO (70×), Český Těšín (69×), S (47×), O-MH (43×), O-P (37×) [13]. The most frequent exceeding of the air concentration limit occurs at the localities V and MCT near the boundary with Poland. 

**Figure 2:**
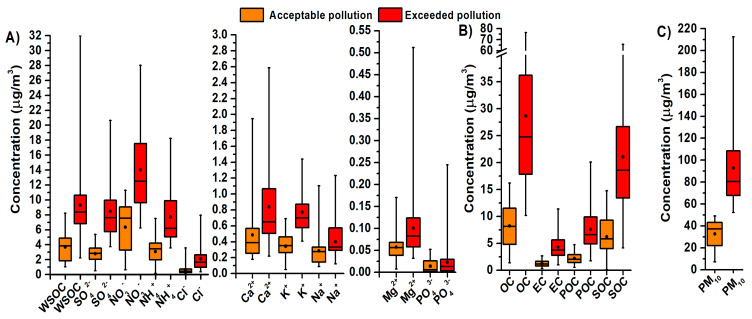
Boxplot in the period of acceptable and exceeded pollution for concentrations of water-soluble ions in PM_10_ (**A**); concentrations of carbon forms in PM_10_ (**B**) and for concentrations of PM_10_ (**C**). Explanations: Water soluble organic carbon (WSOC), secondary organic carbon (SOC), particulate organic carbon (POC).

**Figure 3:**
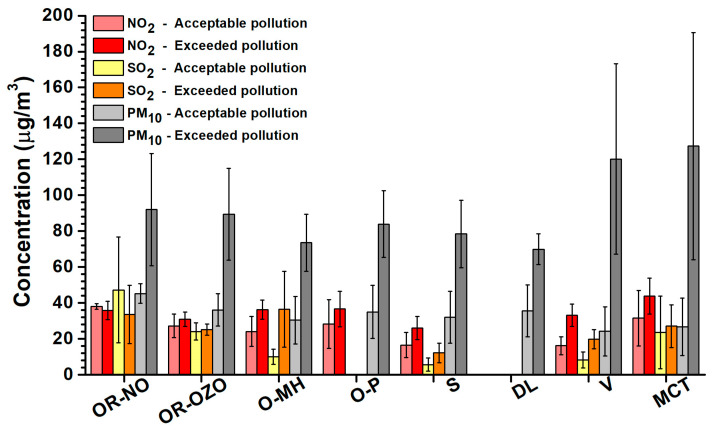
The average concentrations of NO_2_, SO_2_ and PM_10_ with the standard deviations for 2018.

The average concentration of PM_10_ in the period of acceptable pollution was 32.7 ± 13.0 μg/m^3^ (Figure 2). It increased 2.8 times during the period of exceeded pollution 92.8 ± 37.8 μg/m^3^. The highest average concentration of PM_10_ during the period of exceeded pollution was determined in MCT (127 ± 64.4 μg/m^3^) and the lowest in DL (69.9 ± 8.6 μg/m^3^). Average concentrations of NO_2_ and SO_2_ for MSR were during the period of exceeded pollution 1.3 times and 1.5 times higher than in the period of acceptable pollution. Average concentrations of monitored pollutants (NO_2_, SO_2_, and PM_10_) in 2018 for individual localities are illustrated in Figure 3. The locality OR-NO has the values for the period of acceptable pollution higher than other localities. This sampling site is situated in the immediate vicinity of the largest pollution source at the territory of Ostrava, namely the metallurgical enterprise Arcelor Mittal (now Liberty Ostrava). Moreover, it is situated at the morphologically elevated position. The locality OR-NO is situated in the NE direction from the sintering plant that represents the main pollution sources. This direction corresponds to the prevailing air mass flow during sampling period. 

In the period of exceeded pollution, the content of ammonium ions, organic matter and PM_10_ increase significantly (Table 1). The concentration of individual major components in PM_10_ increased differently: up to 3-fold concentration increases occurred in Cl^−^, OM, SO_4_^2−^ and EC, lower than 2-fold increase occurred in NO_3_^−^, NH_4_^+^, K^+^ (Table 1).

Figure 2 shows the boxplots for the main ions represented in inorganic aerosol (IA) and water-soluble organic carbon (WSOC), organic carbon (OC) and elemental carbon (EC) for the period of accetable pollution and exceeded pollution for all measurement.

**Figure 4:**
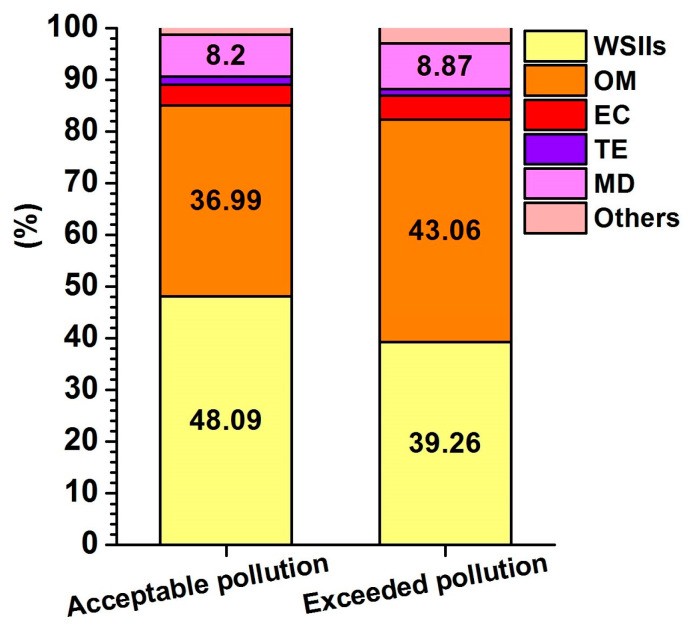
Average composition percentages of PM_10_ during the period of acceptable and exceeded pollution. Explanations: Water soluble inorganic ions (WSIIs), organic matter (OM), elemental carbon (EC), trace elements (TE), mineral dust (MD).

The minimal and maximal average percentage of organic matter in dust particles and water-soluble ions during the period of acceptable pollution (OM: 29.4–48.6% and IA: 53.3–66.8%) and exceeded pollution (OM: 37.3–44.7% and IA: 45.3–55.6%) is almost comparable. The average percentage of PM_10_ is shown in Figure 4. The percentage of inorganic particles (minerals and amorphous particles = mineral dust, MD) was derived by calculation according to Equation (1) by Amato et al. [31]:

(1) MD=Al×3.89+Ca×2.5+Fe×1.43+K×1.21+Ti×1.67

The parameter MD expresses a total (mass) concentration (with certain assumptions regarding the chemical composition of the dust) of insoluble inorganic particles (mineral phases and amorphous inorganic phase). It was used for the construction of Figure 4 in order to obtain a total composition of particles (100%). The origin of elements included in the MD parameter in the Ostrava region is from both blast furnaces and the combustion of fossil fuels.

The average pollutant content in PM_10_ implies that during the period of exceeded pollution, the content of organic substances 43.1 ± 12% is higher by about 7%, which is compensated by the loss of water-soluble ions (Figure 4). Increased OM shares of PM_10_ during smog situations have also been observed in Switzerland [32].

In the region of Ostrava, analyses of organic compounds have shown that the beginning of the smog episode is accompanied by an increase of the percentage of an unresolved complex mixture of hydrocarbons (UCM) and organic matter which cannot be reliably identified. The combustion processes are the most important source of particles PM_10_ and OM during both acceptable pollution and exceeded pollution. The combustion processes include the combustion of coal (lignite, bituminous coal and mixtures of both), biomass and waste (polymers—plastics). Organic compounds of sulphur occurred in all samples of PM_10_ during both acceptable pollution and exceeded pollution in areas with the combustion of coal. The number of organic compounds of sulphur in a deposition during exceeded pollution is two or three times higher than during acceptable pollution. The locality O-MH is characterised by high emissions from transport for both acceptable pollution and exceeded pollution. The locality O-P has similar characteristics. The combustion of biomass prevails among combustion processes. The difference in OM between acceptable pollution and exceeded pollution is not conspicuous. However, individual organic compounds connected with the combustion processes have increased concentrations. During exceeded pollution, EC is not increased despite the fact that it can be expected. This is connected to the fact that the combustion processes play an important role in the winter season even during acceptable pollution (the results of the authors’ team will be published during the year 2020).

### 3.1. Enrichment Factors for the Main Components in PM_10_ in Respect to the Background Values

The aim of determination of enrichment factors (EF_BC_) for the main components was the comparison of their normalized concentrations in PM_10_ with background values. The background values were utilised because Clarke values cannot be used for secondary ions, organic matter, and elemental carbon. Enrichment factors (EF_BC_) for the main components of PM_10_ were calculated according to Equation (2). The concentration of water-soluble ions was normalized to PM_10_ concentrations. The background values were measured at the locality Olomouc. The results are shown in Table 2. The equation for calculation of the enrichment factor in PM_10_:

(2) EFBC=(CxPM10)measured(CxPM10)background

where *C_x_* are the concentrations of the element *x* in PM_10_.

The enrichment factors for sulphates and chlorides in PM_10_ have in the periods of acceptable and exceeded pollution the comparable values. Organic matter has lower EF_BC_ in the period of acceptable pollution compared with the period of exceeded pollution. On the contrary, NH_4_^+^, NO_3_^−^, Ca^2+^_,_ Mg^2+^, K^+^, Na^+^ have different behaviour and their EF_BC_ have relatively lower values in the period of exceeded pollution. The increased concentrations of PM_10_ during the exceeded pollution period result in lower concentrations of these ions. 

Enrichment factors show variability in some parameters, even when comparing individual localities (Table 2). A significantly higher enrichment factor for all monitored parameters except nitrates and ammonium ions was found for OR-NO and OR-OZO in the period of exceeded pollution. The highest enrichment factor for nitrates was found for the locality O-P, which is mainly affected by transport emissions. In the period of acceptable pollution, it also shows the highest enrichment factor for EC. The addition of normalized values shows that in the period of acceptable pollution, Veřňovice (V) belongs to the localities with the highest occurrence of anomalous concentrations. In the period of exceeded pollution, it is Radvanice (OR).

### 3.2. Enrichment Factors of Trace Elements

For trace elements, the enrichment factor was calculated using the standard procedure according to Equation (3):

(3) EFx=(CxCref)PM(CxCref)crust

where C_x_ and C_ref_ are the concentrations of the element *x* in PM_10_, and the reference element, (C_x_/C_ref_)_PM_ and (C_x_/C_ref_)_crust_ are the proportions of these concentrations in PM and in the Earth’s crust, respectively. The concentration of elements in the Earth’s crust (Clarke value) was used in accordance with Wedepohl [33]. Aluminium was used as a reference for comparing EF values with other localities in the Czech Republic (Olomouc). If the EF value ˃ 1, the element is assumed to be relatively enriched in the environment; if EF ˃ 5, the element comes from anthropogenic sources [34], the EF value ranging from 20 to 40 is considered very high, and EF ˃ 40 extremely high [35]. 

**Figure 5:**
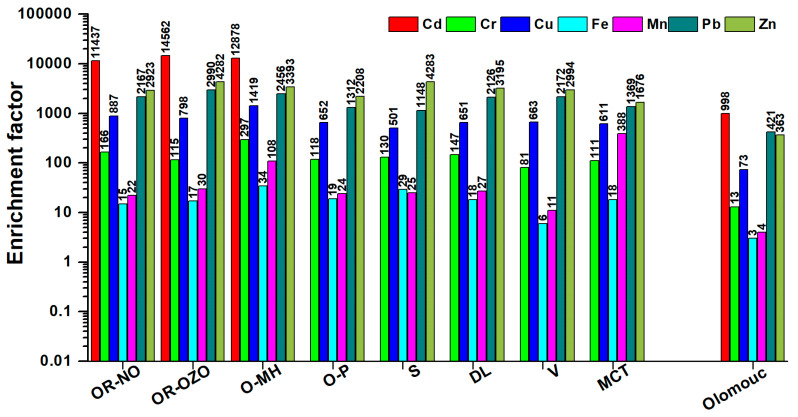
Enrichment factor for trace elements—comparison with Olomouc in the period with acceptable pollution.

Cadmium exhibits an extremely high enrichment factor (14,500) in locations affected by the metallurgical industry OR-OZO, OR-NO, and O-MH. The EF_Cd_ was five times higher than the value found for Olomouc (Figure 5). In other locations, Cd concentrations were below detection. Cadmium is one of the elements usually exhibited by the high enrichment factor ˃1000 [36].

For chromium, the values of the EF in PM_10_ are usually in the range of 10–100 [36]. In MSK sites, EF_Cr_ values vary in the range of 100 to 300, the lowest EF_Cr_ value was found in Olomouc 13. The values of the EF for Cu and Zn are usually around 100 [36]. For sites in MSK, value of EF_Cu_ are lower than the value reported Di Vaio et al. [36] in the range of 10–30, while in Olomouc it reaches a value of up to 70. Transport is considered to be the main source of Cu in PM_10_ particles. EF_Zn_ values in MSK are 20 to 40 times and for Olomouc three times higher than EF_Zn_ according to Di Vaio et al. [36]. The source of Zn may be the burning of fossil fuels, biomass, transport and, in the case of MSK, the metallurgical industry. EF_Pb_ values for in MSK range from 2.0 to 3.0, which is 20–30 times higher than the EF value reported by Di Vaio et al. [36] ˃ 100. A minimum EF of less than 10 is given by Di Vaio et al. [36] for elements of crustal origin Fe (4) and Mn (8). In the case of Fe, the EF in PM_10_ in the MSK region reaches values in the range of 10–13 and for Olomouc 3. EF_Mn_ values show a large range from 10 to 100, except for the MCT site where EF is 800. The background locality Olomouc has an even lower EF_Mn_ in PM_10_ than value published by Di Vaio et al. [36]. The high EF values for Mn are related to the metallurgical industry and the burning of fossil fuels (MCT). The highest values of the acquisition index for all monitored elements were found at the O-MH site affected by the operation of the steelworks, which is in line with the results of Švedová et al. [37]. Average EF values in MSK are EF_Cr_ (145), EF_Cu_ (772), EF_Pb_ (1968), EF_Zn_ (3119), EF_Fe_ (20) and EF_Mn_ (33) clearly show significant impacts on anthropogenic activity, both in relation to the Olomouc background site and in comparison to the data published by Di Vaio et al. [36].

Hazardous trace elements can come from transport tyre wear and brake abrasion: Zn, Cd, Cr, Cu, Ni, Pb [38], Ba, and Zr [39]. In addition to the above-mentioned elements, tyre wear also releases Co, Hg, Mn, Mo, and brake abrasion, Sb [38], Sn [40], Ni, while Cr can also come from the corrosion of cars and chrome plating of some motor vehicle parts [41]. In order to identify pollution from transport, a Cu/Sb 4.8–18.9 and Fe/Cu ratio of 14/40 can be used [40]. Fine particles formed in the combustion processes of fossil fuels also contain hazardous trace elements such as, As, Ba, Be, Cd, Cr, Co, Hg, Pb, Sb, Ni, Zn, and Se [42]. The concentrations of As, Cd, Pb, and Zn in Polish coals are comparable to or greater than those in other European coals [43]. An important source of pollution is the production of Fe and steel in Arcelor Mittal in Krakow and Ostrava. Dust from blast furnaces is a significant source of Fe (13.6%), Ca (5.20%), Zn (1.88%), Pb (0.29%), Na (0.45%), K (0.92%), Cl (2.90%), Cd 72 mg/kg, and Cu 106 mg/kg [44]. The emission factor is determined for the total suspended particulates (TSP). From the results published by Wang et al. [45] and Passant et al. [46] it is clear that in some technologies TSP is mainly composed of PM_10_ (steel making technology: basic oxygen furnace, BOF 85% from TSP are particles below 10 µm and Electric arc furnaces, EAF, 98% particles from TSP is below 10 µm, blast furnace, 88% particles from TSP is below 10 µm). When iron is produced in blast furnaces, dust particles of a minimum of 0.42 and a maximum of 41.95 g/t hot metal (HM) and PM_10_ particles of 0.26–25.92 g/t HM are released into the air [47]. The minimum and maximum emission factor values for Mn 45.12–53.02, Cr 2.76–10.41, Ni 1.99–10.6, Pb 2.19–24.33, and Zn 3.81–12.9 mg/t HM. For the other elements, values are lower in order of As 206–300, Cd 65–223 and Hg 56–200 µg/t HM. Air emission for steel mill according to BAT technologies are related to 1 tonne of steel: dust particles 300 g, PM_10_ 180 g and PM_2.5_ 140 g, Pb 4.6, Zn 4, Cr 4.5, As 0.4, Ni 0.14, Hg 0.1, Cu 0.07, and Cd 0.02 µg/kg [47,48]. Values of emission factors for technologies used in Europe and China are markedly different [47,49]. Results for the iron and steel industry in China [49] provided the highest emission factor for the production of iron in a blast furnace for Zn (890 g/t) followed by V (460 g/t), Mn (376 g/t), Cu (202 g/t), and Pb (1.2 g/t). Emission factors for sintering differ from blast furnaces in the amount of emitted Pb (727 g/t), Zn (582 g/t), Mn (354 g/t), Cu (287 g/t), and V (102 g/t). During the processing of iron in Chinese industry using the electric arc furnace, 35 g Zn, 2.7 g Pb, 1.5 g Cr, and 0.61 g Ni are released per ton of product [49].

It is evident from the emission of hazardous trace elements from transport, iron and steel production and fossil fuels that the identification of the emission sources it may be difficult. The origin of elements included in the MD parameter in the Ostrava region is both from blast furnaces and combustion of fossil fuels.

It is clear from the comparison of EFs in the Olomouc, where pollution from transport and residential heating prevails, that iron metallurgy has a major impact on air pollution. In order to identify pollution sources, a regression analysis was performed for hazardous trace elements. Similarly, relationships between major components of PM_10_ and trace elements were determined. A statistically significant dependence at the level of significance p = 0.005 (for all relationships) was found between Fe and Cr (r = 0.61), Zn (r = 0.76) and Al (r = 0.76), between Al and Cr (r = 0.70), Zn (r = 0.67), and between Zn and Pb (r = 85). Among the major components and trace elements, a statistically significant dependence was found only for Pb × chlorides, Pb × organic matter and Pb × elemental carbon (r = 0.67). The relationship between lead and chlorides can be explained by the presence of mineral phases PbCl(OH) laurionite, Pb_7_F_12_Cl_2_ laurelite and penfieldite Pb_2_Cl_3_(OH), which have been identified in dust from the sintering plant (electrostatic filters) and lithargite (PbO) - fabric filters [50]. Lu et al. [51] identified eight major phases with Pb content in atmospheric aerosols: Pb-Cl, Pb-Cl-Li (coal combustion and waste incineration), Pb-Cl-EC and Pb-Cl-OC (diesel trucks and coal combustion), Pb-Cl-Fe (iron and steel industry), Pb-Cl-AlSi (dust), Pb-Sec (secondary formation), and Pb-Cl-Zn (industrial processes) [51]. A statistically significant correlation coefficient between Pb and EC, OM demonstrates the importance of transport on the region’s air pollution load, and the correlation coefficient between Pb, Cl and Fe demonstrates the emission of iron and steel production in the region.

### 3.3. Cations in Water-Soluble Inorganic Ions of PM_10_

#### 3.3.1. Calcium in PM_10_

Calcium in PM_10_ occurs mainly as a carbonate (calcite and dolomite), usually in quantities of < 10% [52]. Ca-sulphates are also found in PM_10_, including koktaite (NH_4_)_2_Ca(SO_4_)_2_·H_2_O, gypsum CaSO_4_·2H_2_O or bassanite CaSO_4_ [53]. These carbonate minerals are mainly derived from the crust, and a small amount of calcite originate from construction activities [54,55]. Carbonates from building dust may have larger particle sizes than PM_10_ and therefore are not monitored by some authors in PM_10_ [56]. Like ammonia, carbonates may be important for neutralizing acidic gaseous components [57].

It is believed that the water-soluble calcium occurs in the form of chlorides, as confirmed by the high correlation coefficient value (r = 0.75) between chlorides and water-soluble calcium. The average water-soluble Ca^2+^ concentration ranged from 0.26 to 1.75 µg/m^3^ for all localities. In the period of acceptable pollution as well as exceeded pollution, the highest Ca^2+^ concentrations were measured in the locality OR-NO. The highest enrichment factor (EF_clarke_) was found for OR-NO (67), for the locality OR-OZO (47), and in other localities, the value was around 20 except for O-P (9). The EF background for Ca for North-Western European cities (Antwerpen, Amsterdam, Lille, and Leicester), it was 10 [58]. Increased calcium contents in air pollution in municipal district Radvanice, but also in the whole region is related to the production of iron and steel, it was also confirmed by the study of Kozákova et al. [59]. The main mineral in waste dust collected from fabric filters from the sintering plant of the metallurgical enterprise Třinecké železárny is portlandite (Ca(OH)_2_, which represents up to 50% of the dust particulate composition, followed by calcite (CaCO_3_) with 11% and dolomite (Ca, Mg)CO_3_)_2_ with about 6% [50].

#### 3.3.2. Sodium in PM_10_

Increased concentrations of Na^+^ in cities are mainly related to the use of winter spreading materials and the occurrence of marine aerosol in connection with the long-range transboundary transmission, which is very low in Czech Republic < 1% of PM_10_ [60]. The amount of sprinkler material depends on the weather conditions and the type of sprinkler equipment used. The syringes, which use dry or wetted salt for sprinkling, have a smooth dose adjustment between 10 and 60 g/m^2^. The syringes with a scrunching device allow a dose of 5 to 20 g/m^2^ to be used. The recommended concentration of sodium chloride in brine is 18–21% (Highway Code 104/1997 Coll. 23.4.1997 as amended) [61]. Preventive sprinkling is carried out with 5–10 g/m^2^ moistened brine. The precautionary procedure is used to ensure road safety before weather conditions trigger a critical state/downhill.

In 2007, the average value of annual wet deposition (monthly sampling) in the Beskydy Mts. (Červík, 55 km from Ostrava) was for Na 256 mg/m^2^. In 2018, this value decreased to 56 mg/m^2^. An even sharper decrease was observed for chlorides from 938 mg/m^2^ in 2007 to 107 mg/m^2^ in 2018. A stoichiometric recalculation indicates that deposition of 13 mg/m^2^ Na is missing to form NaCl from the observed chloride concentration. Chlorides must be present in another form [62]. 

The mass ratio of Cl^−^/Na^+^ is 4.39 ± 3.48 for the whole set of samples, which confirms the influence of industrial activities on the occurrence of Na in the air aerosol. Similar values for the industrialized regions were reported by Yang et al. [63] and Salam et al. [64]. In the period of acceptable pollution, the highest average concentrations of Na^+^ were identified in the locality OR-NO (706 ± 564 ng/m^3^), in O-P, in the locality affected by traffic load, the concentration was only 376 ± 160 ng/m^3^. In the period of exceeded pollution, the concentrations for the localities OR-NO and O-P were almost equal. The average Na^+^ concentration for the locality OR-NO was 593 ± 128 ng/m^3^ and for the locality O-P 491 ± 314 ng/m^3^. The results of the mineralogical composition of waste dust from electrostatic filters from the sintering plant showed the presence of 12% of NaCl and up to 21% of NaCl in waste dust from fabric filters [65]. An increased concentration of Na in air aerosol mainly results from the production of pig iron and steel [66].

#### 3.3.3. Potassium in PM_10_

The concentrations of potassium in the aerosol are related to the combustion of biomass [67], coal [68] and metallurgical processes [66]. In the case of aerosol in the period of exceeded pollution, sodium represented on average 55.5 ± 38.5% of the total K^+^ content. Multiple sources are involved in the origin of the total concentration of water-soluble potassium. The most significant sources of potassium include the metallurgical industry, where KCl is used for sintering iron ores and can increase the concentration of K^+^ and Cl^−^ in the aerosol. Significant elements released in the production of coke are Ca^2+^, K^+^, and Na^+^ [66] and chlorides [69].

In the period of exceeded pollution, K^+^ has a statistically significant correlation coefficient with sulphates (r = 0.86), chlorides (r = 0.68), ammonium ions (r = 0.85), nitrates, but also organic and elemental carbon. In the period of acceptable pollution, there was no statistically significant dependence between Na^+^ and K^+^. Statistically significant correlation coefficient values with OC and EC confirm that potassium is released during biomass combustion. In order to identify the effect of emissions from biomass combustion, the K^+^/EC ratio value ranging from 0.21 to 0.46 is used to distinguish the origin of EC from fossil fuel combustion (0.025–0.09) according to Andrae (1983), as referred to by [70].

The values of the K^+^/EC ratio for the monitored localities ranged from 0.19 to 0.30, only for locality V, three values were low (0.08–0.09) and corresponded to the combustion of coal. The dependence between chlorides and potassium can correspond not only to biomass combustion but also to metallurgical processes. The high value of EF confirms the impact of iron and steel production processes on the potassium concentration in air pollutants in the locality OR-NO (40). During the period of exceeded pollution and acceptable pollution, higher average values of K^+^ occur in OR-NO (0.95 and 0.58 µg/m^3^) and in locality V (0.94 µg/m^3^).

#### 3.3.4. Ammonium Ions in PM_10_

Gaseous ammonia (NH_3_) is a precursor to inorganic aerosols and is generally the limiting reactant in their formation [71]. For a long time, the main sources of atmospheric NH_3_ were mainly considered to be agricultural activities (microbial decomposition of organic matter and use of fertilizers), which are currently supplemented by transport emissions as a consequence of the use of urea (NH_2_)_2_CO to reduce NO_x_ production in diesel engines. However, conversely, they increase the amount of NH_3_ [24]. An important source of NH_3_ is the combustion of fossil fuels. In urban Beijing, 38–52% of NH_3_ emissions come from burning fossil fuels [23].

Nowadays, technologies for primary NO_x_ control methods used in coal-fired utility boilers utilizing ammonia- and urea-based processes can also contribute to increasing NH_3_ production [72]. Residential combustion represents substantial contributions to NH_3_ emissions in central and southern Europe [73]. Emission factor NH_3_ for industrial coal combustion is 0.015 kg per ton, for coking 0.01 kg per ton, for domestic combustion 0.908 kg per ton and for domestic wood combustion 1.4 kg per ton [74]. The NH_4_^+^ concentration in the background area of Olomouc is 0.296 ± 0.143 µg/m^3^. In the period of acceptable pollution, the highest concentration of ammonium ions (ranging from range from 0.51 to 7.52 µg/m^3^) was found in the locality O-P (average value is 4.22 ± 2.88 µg/m^3^), which is directly affected by transport. During the period of exceeded pollution, the highest concentration of ammonium ions was measured in the locality MCT in the range 5.59–18.24 µg/m^3^ (average value is 10.62 ± 5.18 µg/m^3^). In the period of exceeded pollution, concentrations of ammonium ions are two up to five times higher than during the period of acceptable pollution. The dependence between individual components of water-soluble ions and carbonaceous particles was monitored using linear regression. Statistically significant correlation coefficient values (r ˃ 0.56) at the significance level of 0.005 between NH_4_^+^ and K^+^, Cl^−^, SO_4_^2−^, NO_3_^−^, and the inverse dependence for wind velocity were demonstrated for both investigated periods. In addition, the dependences between the NH_4_^+^ concentration and wind direction and temperature were demonstrated in the period of exceeded pollution. Statistically significant values of the correlation coefficient are supported by the identification of sal ammoniac (NH_4_Cl) mineral phase at all localities except locality S, where sylvite (KCl) was detected at 27% in the crystalline phase. The highest contents of sal ammoniac (34%) in V and in both localities in OR-NO and OR-OZO (31%) were in the crystalline phase. The main crystalline mineral phase (˃70%) is (NH_4_)_4_(NO_3_)_2_SO_4_. The (NH_4_)_5_(NO_3_)_3_SO_4_ only occurred in the locality O-MH in the period of exceeded pollution.

The availability of NH_3_ for the neutralization of acidic components (H_2_SO_4_ and HNO_3_) in the aerosol is determined using the particle neutralization ratio (PNR). Particle neutralization ratio is defined as the ratio of a molar concentration of total ammonium ions to sulphates and nitrates, Equation (4) [75].

(4) $$PNR=\frac{{\left(NH_{4}\right)}^{+}}{\left[2\left(SO_{4}^{2-}\right)+\left(NO_{3}^{-}\right)\right]}$$

If PNR < 1, the ammonia concentration is insufficient, sulphates and nitrates remain acidifying. If PNR = 1, aerosols are considered neutral; required concentration of ammonia is present. If PNR > 1, there is surplus of ammonia to neutralize the acidic sulphates and nitrates completely [76]. PNR values for the period of acceptable pollution ranged between 1.1 (MCT) and 1.46 (O-P), and in the period of exceeded pollution between 1.16 and 1.30. The results of the PNR calculation show that in both periods required NH_3_ gas is available to neutralize both the acidic sulphates and nitrates. Changes in the concentration of nitrates in aerosol do not show a linear dependence in the case of the reduction of the produced gaseous components in the emissions, as is the case with sulphates [77].

The presence of ammonium nitrates is not influenced only by gaseous precursors, but mainly by the presence of sulphate aerosol. Ammonium sulphate is more easily formed than ammonium nitrate [78]. Competition between sulphate and nitrate for available ammonia causes the changes in ammonium nitrate aerosol content [79]. Competition is also affected by the atmospheric conditions: temperature, humidity, and wind speed [72].

### 3.4. Anions in Water-Soluble Inorganic Ions of PM_10_

Concentrations of nitrates are approximately twofold compared to concentrations of sulphates in both periods. During the period of exceeded pollution, but also in the period of acceptable pollution, nitrates were the major anion. The nitrates were also major anion for other localities in Europe as shown in Table 3. The strong correlation dependence between SO_4_^2−^ and Cl^−^ indicates the combustion of coal and lignite [80]. At the same time, the study of Juda-Rezlez et al. [81] confirmed that “sulphate contents in PM during winter are related to substantial SO_2_ emission from coal combustion”. The concentrations of sulphate during the period of acceptable pollution ranged from 0.49 to 5.39µg/m^3^ and in the period of exceeded pollution, the concentration range was varied: from 3.74 to 20.6µg/m^3^ (Table 1). During the period of exceeded pollution, the concentrations of sulphate are higher when we compare with other localities in Europe (Table 3). Sulphate concentrations at individual sites are relatively balanced. Average sulphate concentrations reported for western, central and southern Europe range from 3 to 7 µg/m^3^ [82]. For the background locality Olomouc, the average concentration is 1.23 ± 0.55 µg/m^3^.

The degree of pollution by nitrates and sulphates can be expressed by the ratio (NO_3_^−^/SO_4_^2−^). The value of the ratio (NO_3_^−^/SO_4_^2−^) for slightly polluted air ranges from 0.36 to 0.60 in a less polluted period and from 0.84 to 0.95 for more polluted one [83]. Within the background locality Olomouc, the value of the ratio (NO_3_^−^/SO_4_^2−^) was 1.02.

In the monitored localities, the ratio (NO_3_^−^/SO_4_^2−^) was significantly higher in the range of 1.56–2.12, which is due to the significant predominance of nitrates over sulphates. The lowest ratio values were found for the localities V, M and OR-NO. The concentration ratio value in moles (NO_3_^−^/SO_4_^2−^) for PM concentrations < 50 µg/m^3^ was 1.44–3.15 and for PM concentrations > 50 µg/m^3^ 1.51–1.82. The formation of secondary particles NH_4_NO_3_ is favoured by low temperatures and high humidity (in winter), while the formation of secondary sulphates is supported by photochemical reaction during the warmest month of the year. 

Thus, in the absence of ammonia for neutralization, nitric acid remains free for other reactions. An excess of NH_4_^+^ in the aerosol results in priority formation of NH_4_Cl. Excess nitrates or ammonium ions can be calculated using the Equations (5) and (6) [84]:

(5) $${{NO_{3}}^{-}}_{Excess}=\frac{\left(NO_{3}^{-}\right)}{62}-\left(\frac{\left(NH_{4}^{+}\right)}{18}-\frac{\left(SO_{4}^{2-}\right)}{48}\right)$$

(6) $${NH_{4}^{+}}_{Excess}=\frac{\left(NH_{4}^{+}\right)}{18}-\frac{\left(SO_{4}^{2-}\right)}{48}-\frac{\left(NO_{3}^{-}\right)}{62}$$

For the usual winter period (acceptable pollution), 40% of cases with an excess of NH_4_^+^ were observed, while for the period of exceeded pollution is was 77% of cases of all observed days; these results were confirmed by the presence of sal ammoniac (NH_4_Cl) in PM_10_ at six localities except for S and DL.

Neither in the period of acceptable pollution nor in the period of exceeded pollution, a statistically significant dependence between sulphate content and calcium ions was proven; Ca^2+^ provided statistically significant dependence only with chlorides. Gypsum, basanite, or anhydrite and boussingaultite (NH_4_)_2_Mg(SO_4_)_2_·6(H_2_O) were identified as significant minerals in PM_10_ in the period before 2016. Low sulphate formation capacity was the reason for the verification of sulphate and nitrate production conditions by evaluating the conversion rate of gaseous precursors. Sulphur oxidation ratio (SOR) and nitrogen oxidation ratio (NOR) are used to evaluate the degree of secondary conversion from gaseous precursors as used in e.g., [84]. SOR and NOR are the molar ratios of sulphates and nitrates to the sum of sulphates and SO_2_ or nitrates and NO_2_. Calculations of SOR and NOR were performed according to the equation as used in [84]:

(7) $$SOR=\frac{n\left(SO_{4}^{2-}\right)}{\left[n\left(SO_{4}^{2-}\right)+n\left(SO_{2}\right)\right]}$$

(8) $$NOR=\frac{n\left(NO_{3}^{-}\right)}{\left[n\left(NO_{3}^{-}\right)+n\left(NO_{2}\right)\right]}$$

where *n* means molar concentration. 

The values of SOR higher than 0.1 show that SO_2_ is photochemically oxidized in the atmosphere [85]. The conversion of SO_2_ to sulphate during a haze episode in winter is realised mainly through the aqueous-phase oxidation of SO_2_ by the catalytic oxidation of iron and manganese with weak photochemical activity, low O_3_ concentration, high NO_2_ concentration, and low temperature [86]. 

Due to the low concentration of water-soluble Fe and Mn, the effect of catalytic activity on the oxidation of SO_2_ has not been confirmed [45]. The formation mechanisms of London Fog and Chinese Haze based on outdoor measurements and laboratory simulation were explained by Wang et al. [45] and are generally valid. Their results demonstrate that the aqueous oxidation of SO_2_ by NO_2_ is key to the efficient sulphate formation but is only feasible under two atmospheric conditions: on fine aerosols with high relative humidity and NH_3_ neutralisation or under cloud conditions. In the polluted environment, this SO_2_ oxidation process leads to significant sulphate production rates and promotes the formation of nitrate and organic matter on aqueous particles, exacerbating severe haze development [45]. The optimum conditions for sulphate formation are at low enlightenment, at temperatures below 0 °C, at high RH > 80% when the SOR parameter reaches 0.6, while at RH < 50%, the SOR parameter is below 0.15 [63]. The highest NOR values occur during spring and winter due to favourable conditions for their formation: weaker photo-oxidation capacity, high humidity, and lower O_3_ concentrations [84].

During the period of acceptable pollution, the sulphur oxidation ratio (SOR) reaches the low values with an average of 0.14 ± 0.11, which increases during the period of exceeded pollution to 0.21 ± 0.09. The values of SOR confirm the low ability of sulphate formation, according to [63]. The average values of NOR in MSR during the period of acceptable pollution (0.17 ± 0.07) and exceeded pollution (0.25 ± 0.07) indicate the more intense secondary formation of nitrate in the period of exceeded pollution than in the period of acceptable pollution. A significant correlation relationship was found between NOR and O_3_ (r = −0.70). At the same time, a significant correlation was found between relative air humidity and both SOR (r = 0.74) and NOR (r = 0.90). The SOR and NOR exhibited an inverse correlation relationship with the temperature (r = −0.67 and r = −0.65 respectively). A similar dependence is reported by Zhao et al. [87].

**Figure 6:**
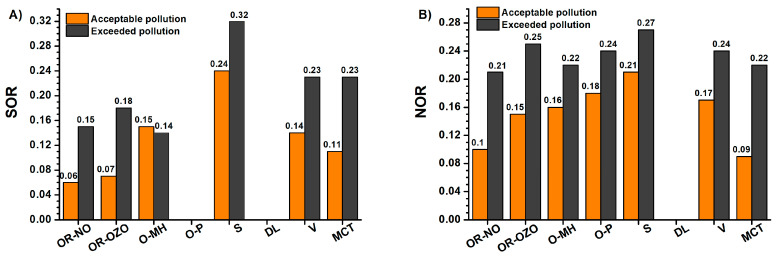
(**A**) Average values of sulphur oxidation ratio (SOR) and (**B**) nitrogen oxidation ratio (NOR) for acceptable pollution and exceeded pollution.

During the period of exceeded pollution, there is an increase of NOR in all localities (Figure 6) approximately 1.7 times. The most significant increase (2.4 times) was recorded in the locality MCT. During the period of exceeded pollution, the average increase in SOR is 1.8 times. 

Nitrates range between 0.63 μg/m^3^ to 11.3 μg/m^3^ in the period of acceptable pollution, and in the period of exceeded pollution, their concentrations increase up to 6.26–28.0 μg/m^3^. The highest average nitrate concentrations in the period of acceptable pollution were found for the locality O-P (8.3 ± 4.4 µg/m^3^), where concentrations are affected by traffic. In the period of exceeded pollution, the highest average concentrations were measured in the localities of MCT (17.8 ± 8.6 µg/m^3^) and V (14.6 ± 6.7 µg/m^3^), localities affected mainly by residential heating. The nitrate concentration in the background locality in Olomouc was 1.25 ± 0.52 µg/m^3^ (Table 3).

Primarily, chlorides are a significant component of aerosol in the marine environment [88]. They are released as a result of various anthropogenic activities (coal and biomass combustion, hydrochloric acid production, paper production, chlorine-containing plastics). Chlorides are an important part of the dust particles released in the sintering of Fe-ores, which can contain up to 30% [89]. The origin of chlorides from biomass combustion is determined by the correlation dependences between potassium and levoglucosan (thermal decomposition product of starch and cellulose) [90], chloride with levoglucosan (r = 0.58, n = 66, α = 0.05), and chloride with potassium (r = 0.70, n = 68, α = 0.05). In the period of acceptable pollution, the highest chloride concentration in IA was measured in the locality OR-NO with an average of 2.46 ± 1.58 μg/m^3^. The highest average concentration was found in the locality V (3.09 ± 2.05 μg/m^3^) and MCT (3.06 ± 2.83 μg/m^3^). 

The annual average background chloride concentration for Olomouc is 0.34 ± 0.14 μg/m^3^. The maximum chloride concentrations in the localities V and M are about nine times higher than the background value in Olomouc (Table 3).

In the period of acceptable pollution, a statistically significant dependence between Cl^−^ and NH_4_^+^, Na^+^, K^+^, EC, SO_4_^2−^, SO_2_, and NO_X_ was found, and in the period of exceeded pollution, a dependence was also found between NO_3_^−^, PO_4_^3−^, and OM (r ˃ 0.62). Linear regression analysis with levoglucosan was performed to identify biomass combustion. Levoglucosan provided statistically significant correlation coefficient values not only for sulphates (r = 0.92), but also for potassium (r = 0.68) and chlorides (r = 0.80). Chlorides come from both coal combustion [91] and biomass combustion [92]. The study Yudovich and Ketris [93] said that world average Cl contents in coals (coal Clarke of Cl) for hard and brown coals are, respectively, 340 ± 40 and 120 ± 20 mg/kg. Moreover, the study by Jagustyn et al. [94] notes, that content of chlorine in wood biomass amounts from below 0.005% to 0.057%. In the case of energy crops, the content of chlorine can even reach 1.0%.

### 3.5. Carbonaceous Particles of PM_10_

Carbonaceous species, organic carbon (OC) and elemental carbon (EC), constitute a major, sometimes dominant, fraction of atmospheric particulate matter [102]. Elemental carbon is released into the atmosphere solely as a product of primary origin from the incomplete combustion of fossil fuels and biomass, but also from transport [103]. EC has a graphite-like microcrystalline structure, is refractory and strongly light-absorptive, which influenced enhancing the hydrophilicity of soot particles in PM_10_ with major environmental effects [104]. Organic carbon is emitted into the atmosphere from anthropogenic or biogenic sources such as primary organic carbon (POC), or it may be of secondary origin (SOC). Secondary organic carbon is formed by condensation of organic compounds formed during photochemical reactions in the air, conversion of gas-particle from volatile organic compounds with low vapour pressure, physical and chemical adsorption [105]. The amount of organic matter in PM_10_ is calculated from the OC concentration by multiplying by the factor 1.4. The OC/EC ratio is used for classifying different sources [106]. At the background locality Olomouc, the average OC concentration is 2.41 ± 1.08µg/m^3^, and the winter concentration is about 30% higher. The average annual EC concentration is 0.61 ± 0.34 µg/m^3^. Winter concentration is also about approximately 30% higher. The OC/EC ratio for winter is 7.5, which is within the range for residential raw-coal combustion in China: OC/EC = 2.5–10 [107]. The OC concentration during the period of exceeded pollution is higher when we compare with other parts in Europe (Table 3). The variability of OC content in PM_10_ is significant during the period of acceptable and exceeded pollution. During the period of exceeded pollution, the OC concentration is higher (2.6 to 6.4 times) compared to the period of acceptable pollution (Figure 2). The lowest differences in organic matter concentration during the period of acceptable and exceeded pollution were found for the localities DL, S and O-MH. The average OC concentration during the period of acceptable pollution was 8.24 ± 4.54 μg/m^3^ (24.4 ± 8.41% from PM_10_), the average organic matter concentration (OM) was 10.9 ± 5.86 μg/m^3^ (32.5 ± 10.6% from PM_10_) and EC 1.26 ± 0.63 μg/m^3^ (3.91 ± 1.28% from PM_10_). The average OC concentration for the period of exceeded pollution reaches the value of 28.7 ± 13.4 μg/m^3^ (30.8 ± 6.95% from PM_10_), for OM 39.1 ± 18.5 μg/m^3^ (41.7 ± 7.94% from PM_10_), for EC 4.30 ± 2.25 μg/m^3^ (4.58 ± 1.51% from PM_10_). In the analysed group, the average OC/EC value ranges from 6.37 to 7.90, which corresponds to the burning of fossil fuels. When comparing the average concentration of OC and EC in the period of acceptable pollution with the background value in Olomouc, the increase is 3.4, and for EC, it is 2.1. In the period of exceeded pollution, the increase for OC is 11.9× and for EC 7.2×. Significant Spearman correlation dependence between EC, OC and wind speed, relative humidity, sulphates, nitrates, ammonium ions, and potassium were found during the period of acceptable pollution. For EC, a statistically significant dependence with chlorides was proven, which shows the effect of combustion processes. In the period of exceeded pollution, there were also statistically significant dependencies of the direction of wind and concentrations of chlorides.

### 3.6. Impact Evaluation of the Meteorological Condition

**Figure 7:**
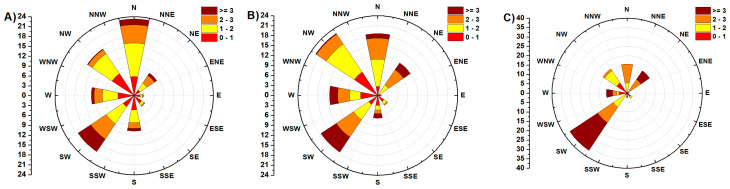
The wind rose for O-P: (**A**) years 2012–2018; (**B**) year 2018 and (**C**) December 2017–February 2018.

**Figure 8:**
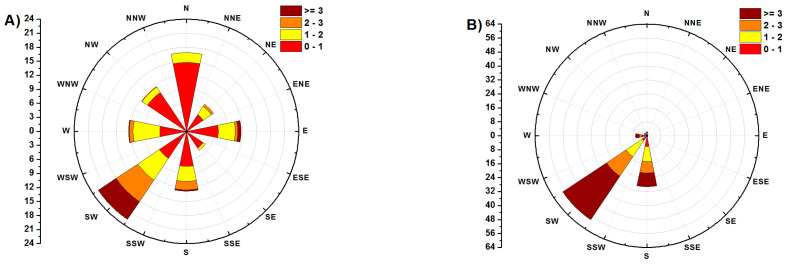
The wind rose for MSR for (**A**) Exceeded pollution, (**B**) Acceptable pollution.

A predominant airflow of “relatively cleaner air from less polluted areas of the Czech Republic” from the southwest is typical of the area of northeast Moravia in the Czech Republic and is related to the orographic influence of the Moravian Gate (Figure 7 and Figure 8). Conversely, northeast and variable wind mass flow with low wind velocities are associated with anticyclonic situations (high-pressure systems), and they are often accompanied by deteriorated dispersion conditions, especially during the cold period of the year. Generally, during predominantly good dispersion conditions, the pollutants are usually transported from the Czech Republic into Poland, whilst during predominantly worsened dispersion conditions, it is the opposite [17].

**Figure 9:**
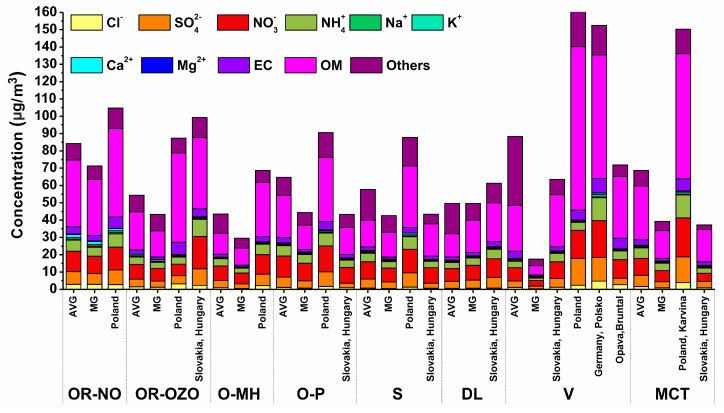
The composition of PM_10_ during different air flowing (MG—Moravian Gate) for all measurements, AVG—average.

Exploratory data analysis (EDA) was performed to evaluate the similarity of individual localities in terms of air pollution load. When using cluster analysis, the collected PM_10_ samples were divided according to the airflow direction. This division has proven to be essential since it affects the amount of PM_10_ and thus its composition. Differences in the amount and composition of PM_10_ for different wind directions are shown in Figure 9.

**Figure 10:**
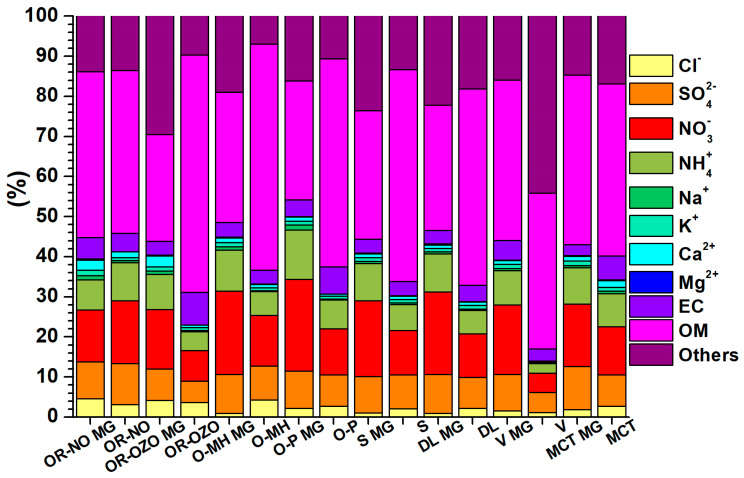
The chemical composition of PM_10_ particles (%) for airflow from Moravian Gate (MG) and Poland.

In addition to the airflow from Poland, the direction of flow from Hungary through Slovakia is also significant, as is the direction from the Ruhr area (Germany) through Poland. Air mass flow was obtained from a database of the Czech Hydrometeorological Institute and calculated using the HYSPLIT modelling system for simple air particle trajectories [108]. The average composition of PM_10_ in the days when all localities were affected by the same wind direction (204–232) from MG is in Figure 10. In these climatic conditions, the localities of V, MCT, S and DL may be considered background localities. Similarly, the contribution from transport in the locality O-P can be calculated by comparison with the background value S (23%) of PM_10_. The difference in concentrations shows that at most localities, in the case of airflow from Poland, the concentrations of water-soluble ions decreased by 12.86 ± 8.31% and the content of “others”, which mainly represents resuspension particles and amorphous fly ash particles formed by silicates or Fe-oxide matrix by 7.21 ± 5.50%. The exception is the locality V, where the share of “others” increased by about 28%. The EC content increased by 1.04 ± 0.56%, the organic matter content by 14.50 ± 10.03%. Increasing the content of OM and EC carbonaceous particles at the expense of WSIIs may pose significant health risks when carried into the respiratory system from inhalation of particulates because they can contain high concentrations of polycyclic aromatic hydrocarbons [109]. Heavy metals can act as a catalyst to stimulate the formation of secondary ions [110]. The comparison of the share of ion concentrations in airflow from Poland and airflow from Moravian Gate shows that in the case of airflow from Poland, in the locality OR-NO, which is affected by the production of iron and steel in Arcelor Mittal, there is no increase in the concentration of Cl^−^, Na^+^, K^+^, and Ca^2+^ produced within the production technology at a higher rate than is carried by long-distance transport (Figure 10). The highest enrichment was found in chlorides (2.13–12.88) in O-MH. Sulphates are enriched 1.7–3.6 times (V), ammonium ions and nitrates exhibit lower enrichment (1.27–2.02). Enrichment of these ions is related to the combustion of fossil fuels. Significant is the increase in organic compounds, which ranges from 1.58–6.71 for organic matter with an average of 3.53. In order to identify the influence of Arcelor Mittal in the locality OR-NO, the concentration values measured during airflow from Moravian Gate in the locality OR-OZO were selected. The difference between the concentrations of PM_10_, ions and EC, OC at both sites shows that Arcelor Mittal contributes to increasing the concentrations of K^+^, Ca^2+^ (1.2–3%), Cl^−^ (5–6%), EC and NH_4_^+^ (6–7%), SO_4_^2−^ and NO_3_^−^ (10–11%), and OM (48–53%).

## 4. Conclusions

The influence of iron and steel production in the Silesia region (both Czech and Polish part) is reflected in a significant increase in enrichment factor (Clarke value). Enrichment factor for Cd (15,000) is enormously high, about three times higher than the value calculated in North-Western European cities and five times higher than in Olomouc. Significantly higher values of EF were found for Cu (1420), Pb (2990), and Zn (4282), which is 20 times more Cu than for the background locality Olomouc, seven times more Pb and 12 times more Zn. The influence of Fe and steel production was demonstrated by the presence of Pb-Cl-OC particles in air concentrations.

The use of EDA (cluster analysis) for the analysed sample showed that the analysed samples are grouped according to the direction of the wind flow, which in the case of the airflow from Moravian Gate shows very low PM_10_ air pollution. On the contrary, wind from the NE direction is primarily due to the high air pollution load, which is characterized by an increased proportion of OC (up to 14.5 ± 10.3%) at the expense of decreasing WSIIs. An increase in OC share represents a higher health risk due to PAHs and trace elements binding.

By comparing the background concentration in the area (comparison of OR-NO and OR-OZO), the share of Arcelor Mittal Ostrava (now Liberty Ostrava), which is 45 ± 8 µg/m^3^ under optimal dispersion conditions, was identified. Some elements of WSIIs, Na^+^, Ca^2+^, and partly also K^+^ and Cl^−^, which are also bound to other processes (biomass combustion), can be considered as indicator elements, showing the significance of pollution from iron and steel production.

## Figures and Tables

**Table 1:** The minimal and maximal concentration ranges of all measured values, arithmetical means (AVG), standard deviations (STD) and ratio of average values AP/EP for the period of acceptable (AP) and exceeded pollution (EP).

Mass Concentration	Acceptable Pollution (AP)		Exceeded Pollution (EP)		EP/AP
	AVG ± STD	Min–Max	AVG ± STD	Min—Max	
	(µg/m^3^)	(µg/m^3^)	(µg/m^3^)	(µg/m^3^)	
Cl^−^	0.66 ± 0.69	0.11–3.57	2.13 ± 1.67	0.37–7.96	3.21
SO_4_^2^^−^	2.79 ± 1.31	0.49–5.39	8.46 ± 3.44	3.74–20.6	3.03
PO_4_^3^^−^	0.014 ± 0.017	0.003–0.05	0.02 ± 0.04	0.001–0.25	1.64
NO_3_^−^	6.36 ± 3.26	0.63–11.30	14.0 ± 5.43	6.26–28.0	2.21
NH_4_^+^	3.07 ± 1.65	0.17–7.52	7.72 ± 3.47	3.59–18.2	2.51
Na^+^	0.29 ± 0.20	0.09–1.11	0.40 ± 0.20	0.12–1.23	1.40
K^+^	0.34 ± 0.17	0.05- 0.69	0.77 ± 0.27	0.41–1.44	2.24
Ca^2+^	0.49 ± 0.35	0.18–1.95	0.84 ± 0.55	0.22–2.59	1.72
Mg^2+^	0.06 ± 0.03	0.01–0.17	0.10 ± 0.07	0.03–0.51	1.75
WSOC	3.67 ± 1.79	1.05–8.24	9.30 ± 4.61	2.25 – 32.0	0.84
EC	1.26 ± 0.63	0.30–2.69	4.30 ± 2.25	1.00–11.4	3.42
OM	10.9 ± 5.86	1.96–21.4	39.1 ± 18.5	14.3–107	3.59
PM_10_	32.7 ± 13.0	7.15–49.0	92.8 ± 37.8	52.4–212	2.84

**Table 2:** Enrichment factors for the period of acceptable and exceeded pollution using the background values of Olomouc.

Localities	Cl^−^	SO_4_^2^^−^	NO_3_^−^	NH_4_^+^	Na^+^	K^+^	Ca^2+^	Mg^2+^	EC	OM
	Acceptable pollution (AP)									
OR-NO	2.14	0.92	1.30	3.70	0.69	1.30	1.96	0.78	1.14	0.99
OR-OZO	1.14	0.88	1.81	3.81	0.27	1.01	1.30	0.68	0.83	1.29
O-MH	0.57	0.99	2.24	4.34	0.23	1.05	0.53	0.32	0.69	1.33
O-P	0.78	0.86	2.32	5.16	0.60	1.02	0.69	0.57	1.04	1.24
S	0.48	0.74	1.97	3.48	0.43	1.02	0.93	0.64	0.84	1.41
DL	0.45	0.93	2.13	4.06	0.34	0.94	0.58	0.53	0.70	1.31
V	0.41	0.55	1.18	2.13	0.71	0.58	0.88	0.39	0.44	0.73
MCT	0.87	1.06	1.63	3.52	0.52	1.30	0.80	0.64	1.15	1.91
AVG-AP	0.88	0.92	1.91	3.96	0.46	1.08	0.96	0.59	0.92	1.37
	Exceeded pollution (EP)									
OR-NO	1.23	0.94	1.51	3.38	0.27	1.17	1.14	0.74	1.11	1.91
OR-OZO	1.06	0.85	1.53	3.29	0.13	0.75	0.52	0.26	1.18	1.89
O-MH	1.09	1.07	1.87	4.36	0.12	0.78	0.44	0.25	1.05	1.68
O-P	0.67	1.03	1.89	3.85	0.18	0.88	0.37	0.28	1.04	1.57
S	0.50	1.02	1.81	3.94	0.19	0.96	0.65	0.40	0.73	1.51
DL	0.54	1.08	1.64	3.67	0.20	0.96	0.47	0.41	0.96	1.61
V	0.86	0.68	1.14	2.77	0.13	0.66	0.27	0.18	0.98	1.55
MCT	0.94	1.00	1.48	3.76	0.16	0.81	0.39	0.34	0.97	1.75
AVG-EP	0.87	0.96	1.61	3.65	0.17	0.88	0.53	0.36	1.01	1.70

Explanations: AVG-AP the arithmetical means for acceptable pollution; AVG-EP the arithmetical means for exceeded pollution.

**Table 3:** Comparison of concentrations of inorganic aerosols and other water-soluble ions in dust particles in the atmosphere of European cities (µg/m^3^).

Location	Period of Sampling	PM_10_	EC	OC	SO_4_^2^^−^	NO_3_^−^	NH_4_^+^	Cl^−^	Na^+^	K^+^	Ca^2+^	Mg^2+^	References
Poland	2007	11.99	-	-	2.41	2.06	1.1	-	0.12	0.03	0.03	1.10	[95]
United Kingdom	2007	11.94	-	-	1.53	1.87	0.65	-	1.81	0.60	0.70	0.16	
Zabrze (Poland); urban background	8–12 2008	-	-	-	1.93	1.05	0.96	0.78	0.27	0.19	0.35	0.79	[96]
Zagreb (Croatia); residential-industrial-traffic site	2006	-	-	-	-	-	-	0.16	0.30	0.12	0.14	0.05	[97]
Melpitz (Germany); rural background	Winter 2004–2008	-	-	-	-	-	-	0.57	0.43	0.18	0.11	0.07	[98]
Venice, Italy	Winter 2006	69.00	-	-	3.63	10.93	3.68	-	-	1.02	1.35	0.16	[99]
Praha-Libuš (Czech Republic)	Winter 04.2008–03.2009	26.68	1.59	5.99	2.86	3.15	1.67	0.22	0.16	0.13	0.22	0.03	[100]
Augsburg (Germany)	Winter (14.11.2007–31.3.2008)	42.70	4.44	4.182	2.285	6.77	2.515	1.836	1.19				[101]
OR-NO	Average concentration– Acceptable pollution	45.23	2.34	8.16	3.87	5.56	3.73	2.45	0.71	0.58	1.45	0.11	This study
OR-OZO		36.09	1.29	9.58	2.80	6.51	3.27	1.20	0.23	0.38	0.88	0.09	
O-MH		30.35	0.98	8.14	2.87	6.50	3.06	0.42	0.15	0.31	0.26	0.03	
O-P		35.00	1.61	7.74	2.64	8.32	4.22	0.51	0.38	0.37	0.43	0.06	
S		32.02	1.13	7.85	2.43	6.57	2.82	0.37	0.26	0.31	0.42	0.06	
DL		35.46	1.16	8.36	3.18	7.13	3.25	0.40	0.26	0.32	0.33	0.06	
V		24.13	0.87	5.88	1.99	4.88	2.08	0.37	0.35	0.26	0.36	0.04	
MCT		26.66	1.39	9.25	2.61	4.10	2.09	0.59	0.31	0.34	0.35	0.05	
OR-NO	Average concentration– Exceeded pollution	92.01	4.56	29.40	8.16	13.21	7.15	2.84	0.59	0.95	1.76	0.20	
OR-OZO		89.38	4.07	29.32	8.30	13.91	7.50	2.35	0.28	0.61	0.72	0.06	
O-MH		73.49	3.63	21.20	7.44	13.53	7.61	1.85	0.20	0.57	0.51	0.05	
O-P		83.93	4.12	25.74	8.26	15.04	7.57	1.56	0.49	0.72	0.57	0.08	
S		78.40	2.72	20.91	7.54	13.18	6.94	0.96	0.34	0.71	0.85	0.09	
DL		69.85	3.18	21.27	7.23	11.14	5.94	0.86	0.33	0.66	0.54	0.09	
V		120.1	6.05	37.66	9.06	14.63	8.70	3.09	0.39	0.88	0.63	0.07	
MCT		127.3	5.59	41.14	11.80	17.82	10.62	3.06	0.44	0.94	0.67	0.11	
Olomouc	November 2018	13.27	0.6	3.37	1.23	1.25	0.30	0.34	0.30	0.13	0.22	0.04	

## Supplementary Materials

The following are available online at https://www.mdpi.com/1660-4601/17/10/3447/s1, Figure S1: The figures of the sampling site (without locality DL) and their location on the map, Table S1: Characteristics of the localities.
