# Supplementary material for: Concentration Variability of Water-Soluble Ions during the Acceptable and Exceeded Pollution in an Industrial Region
Source: Int J Environ Res Public Health. 2020 May 15;17(10):3447. doi: 10.3390/ijerph17103447 (PMC7277652; doi:10.3390/ijerph17103447)
Supplement: Supplementary file 1 [file ijerph-17-03447-s001.zip › Supplementary Material 2.docx]

**Supplementary Material 2**

The figures of the sampling site (without locality DL) and their location on the map.

| 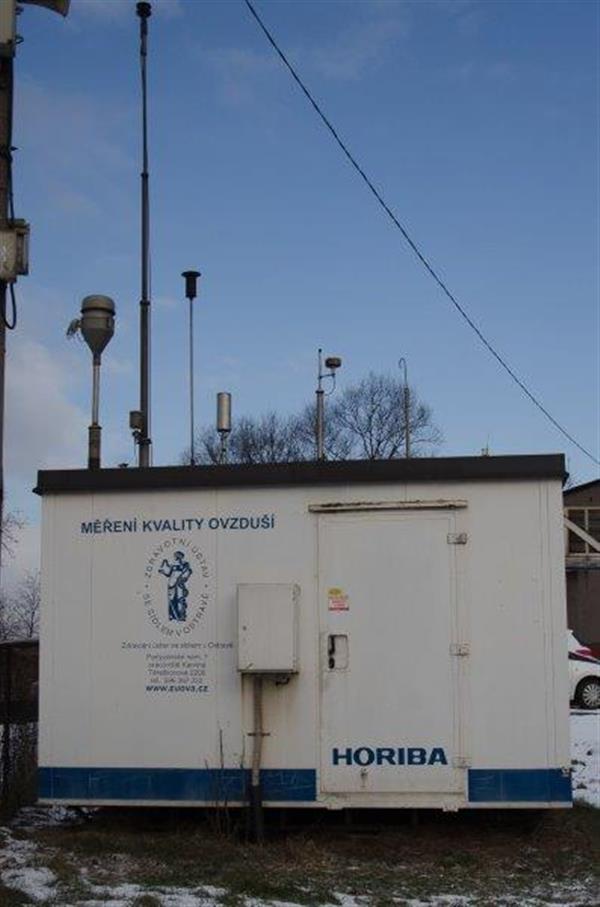  OR-NO [89] | 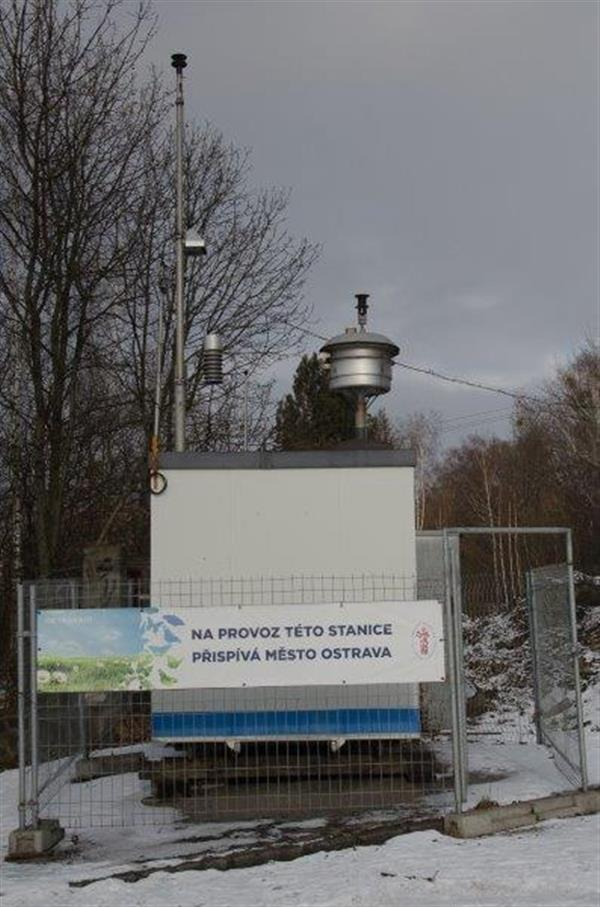  OR-OZO[89] |
| --- | --- |
| 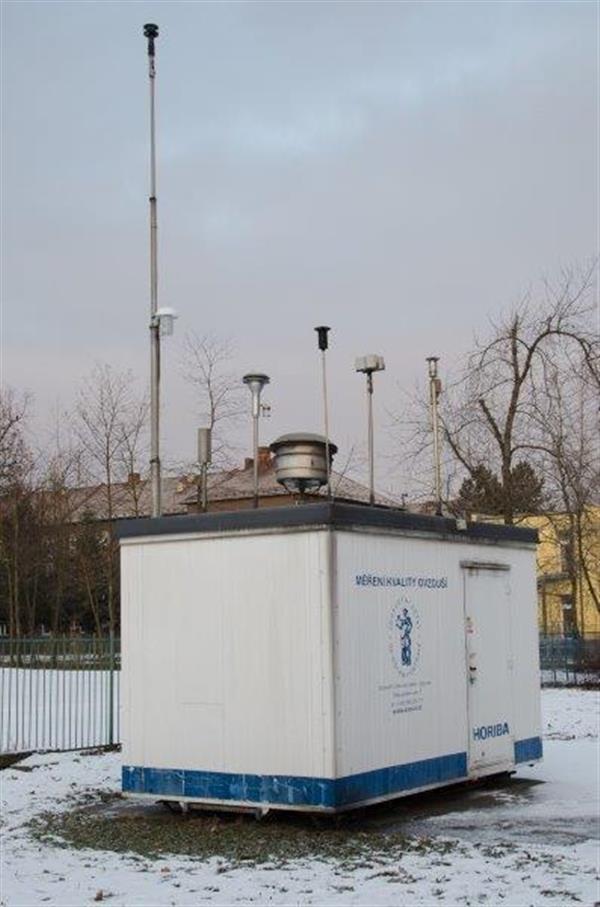  O-MH [89] | 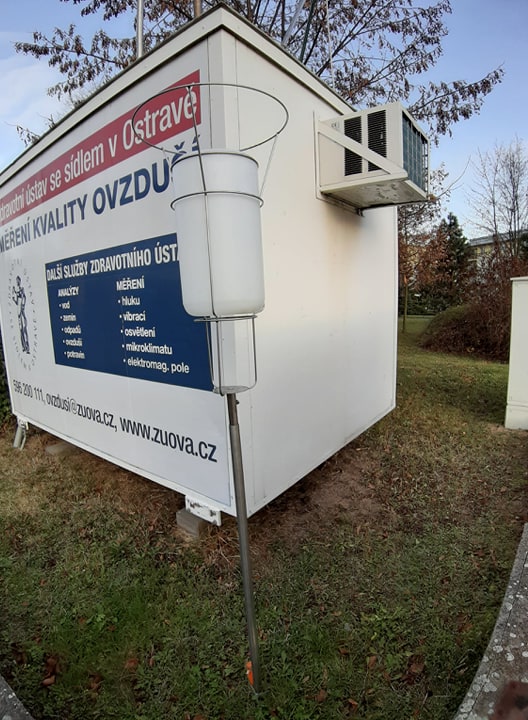  O-P [*] |
| 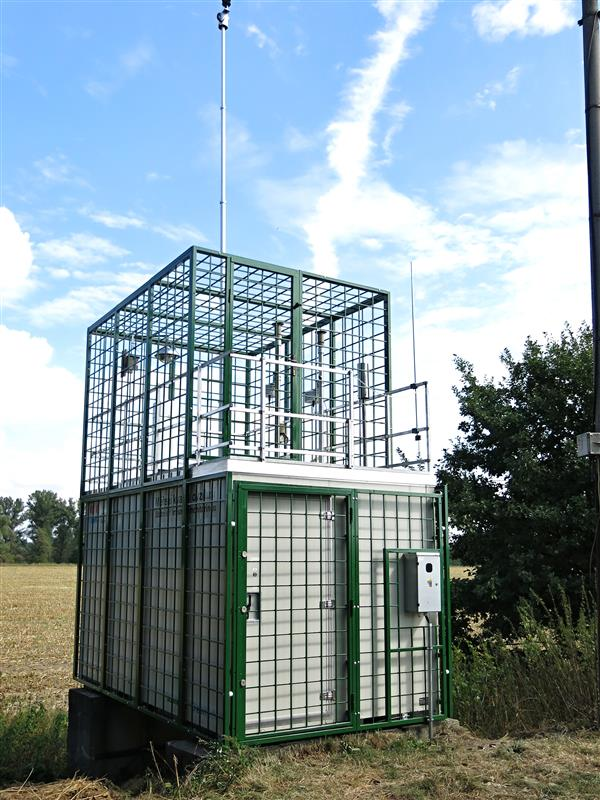  V [89] | 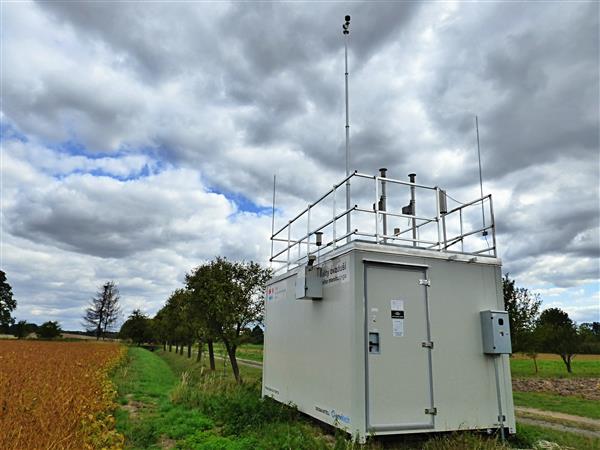  S [89] |
|   MCT [*] |   Olomouc [*] |

Source of the figures:

1. Information on air quality in the Czech Republic, http://portal.chmi.cz/files/portal/docs/uoco/web_generator/tab_reports/automated/tab_2020_01_1M_CZ.html (Accessed: 20-March-2020), In Czech.

* Photographs taken by the authors

| 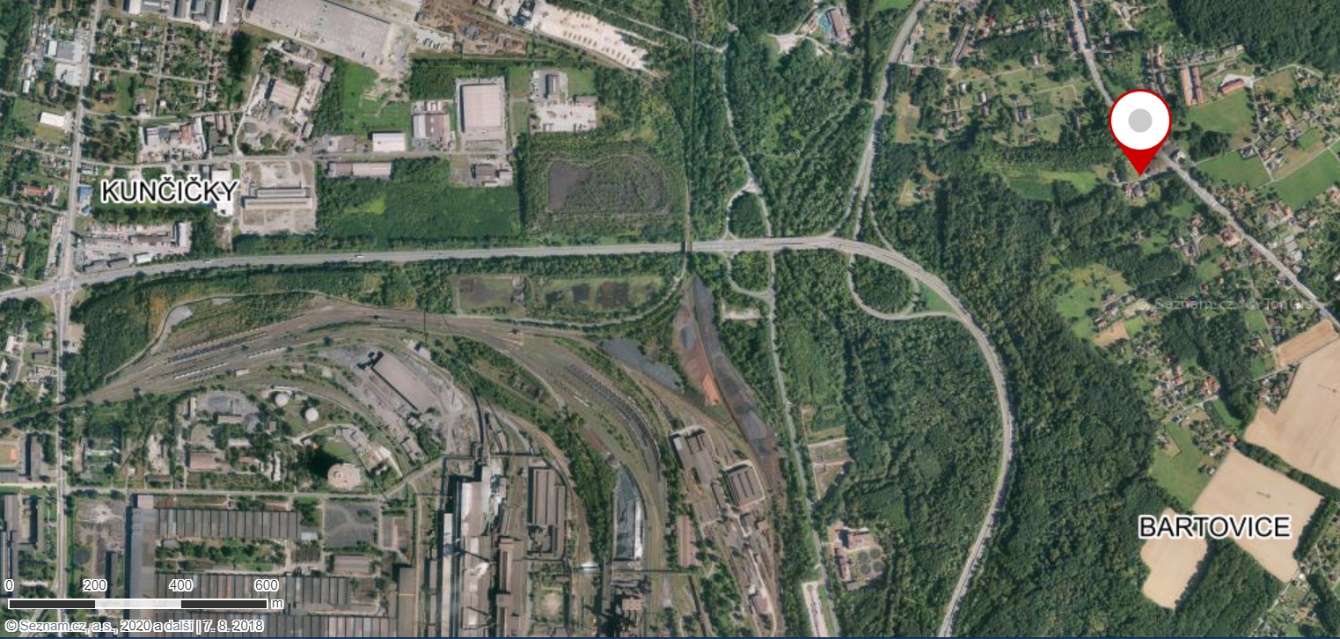  OR-NO [90] |
| --- |
| 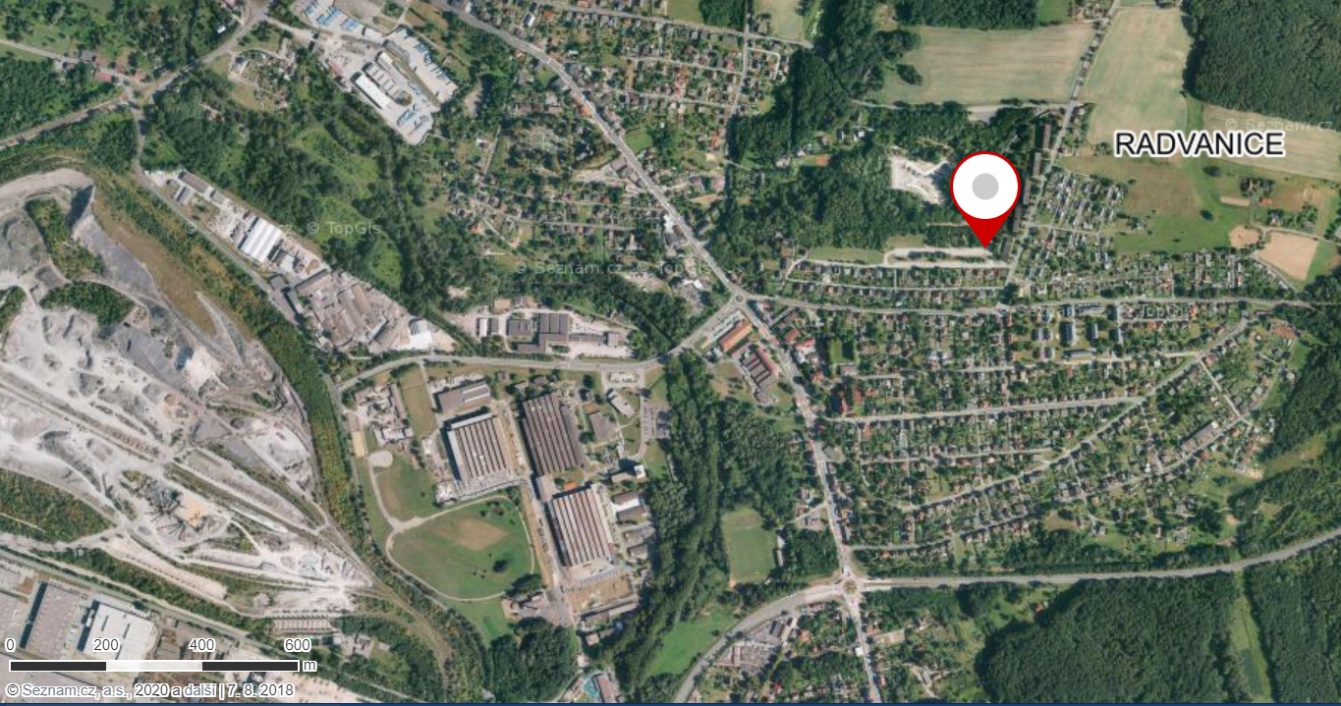  OR-OZO [90] |
| 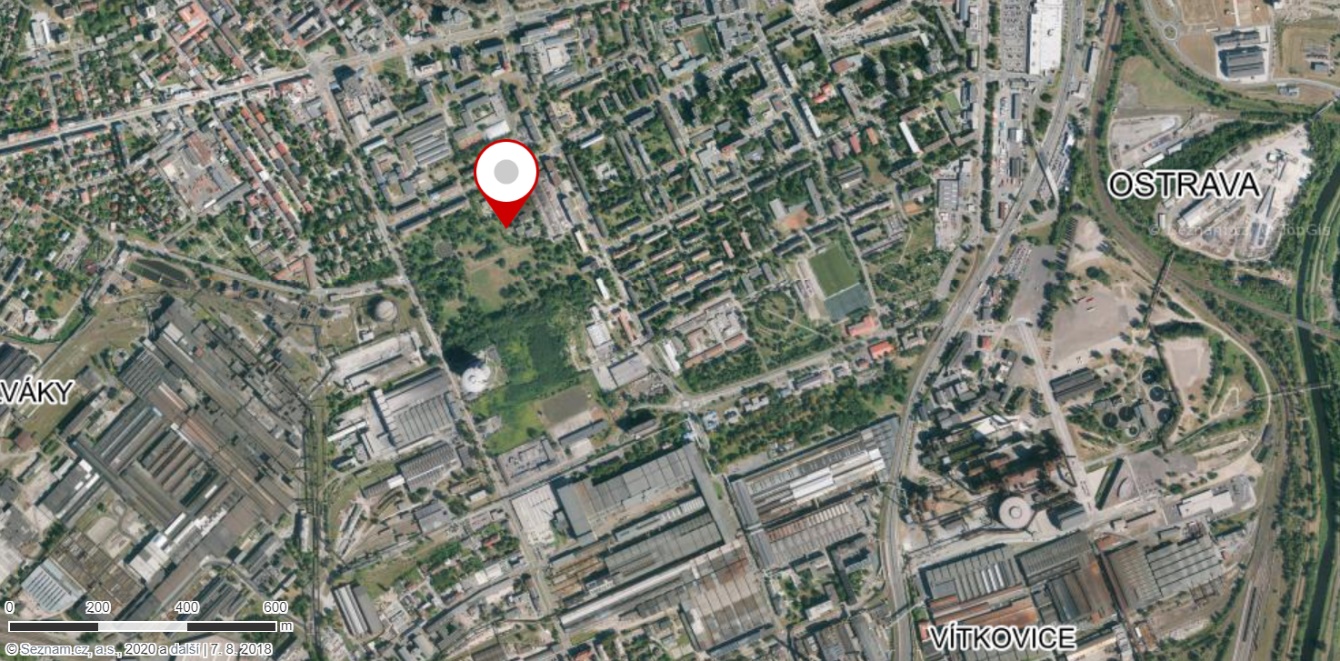  O-MH [90] |
| 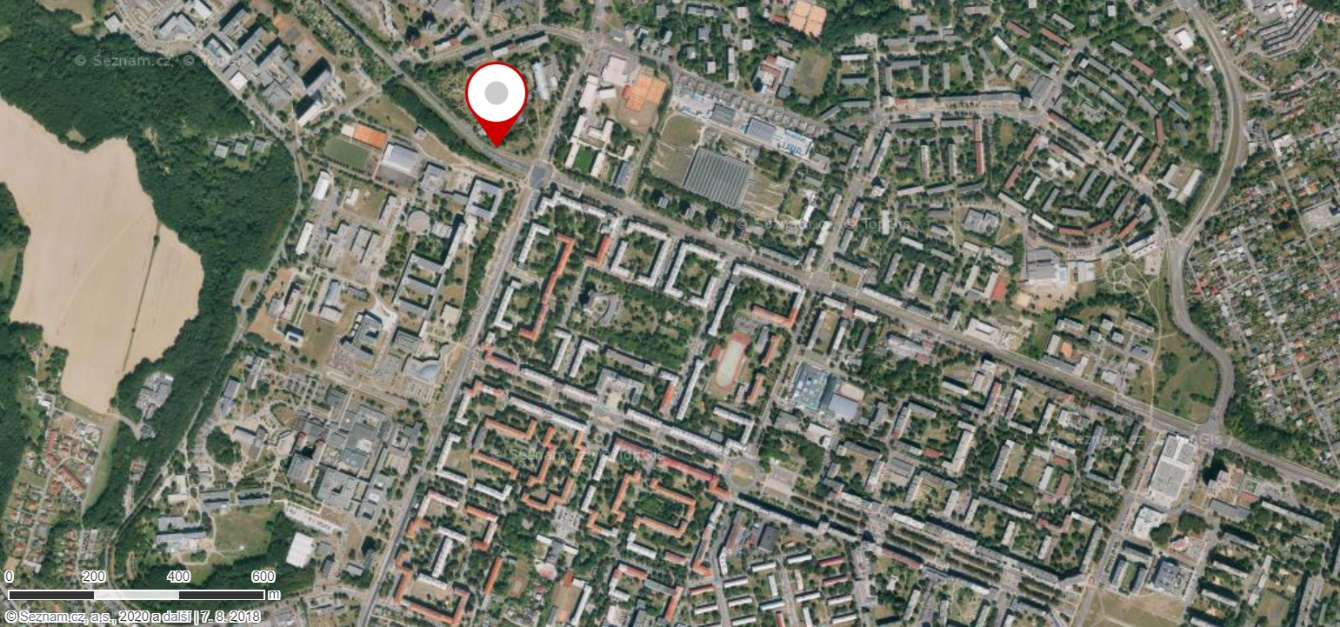  O-P [90] |
| 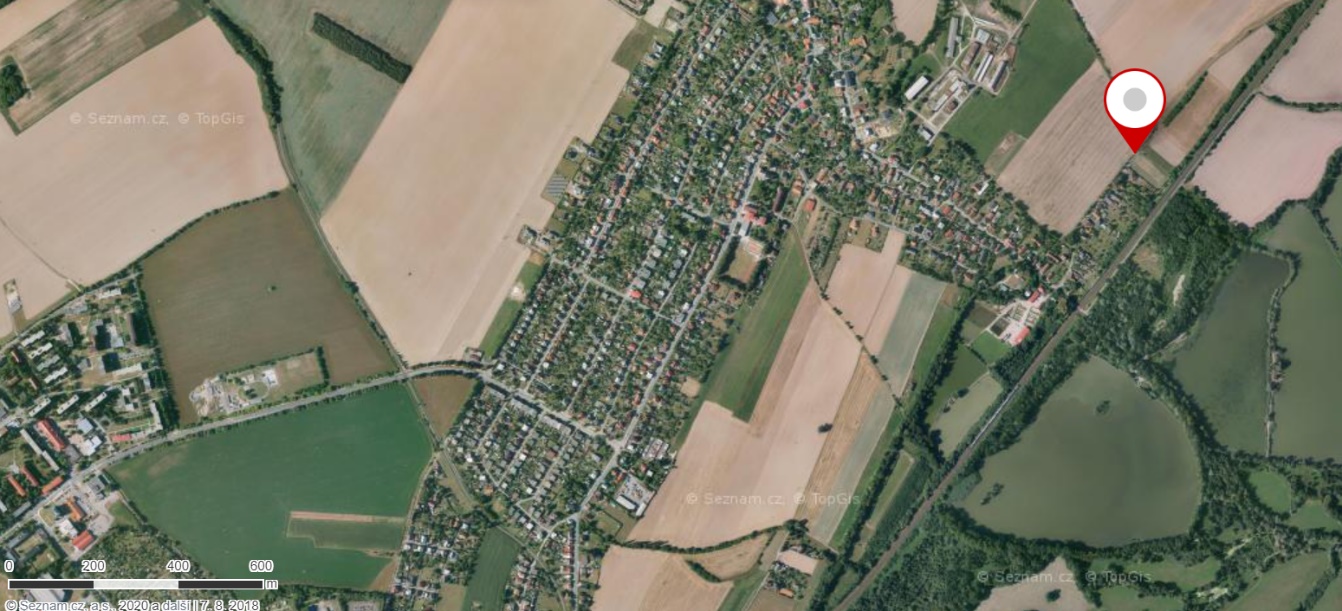  S [90] |
| 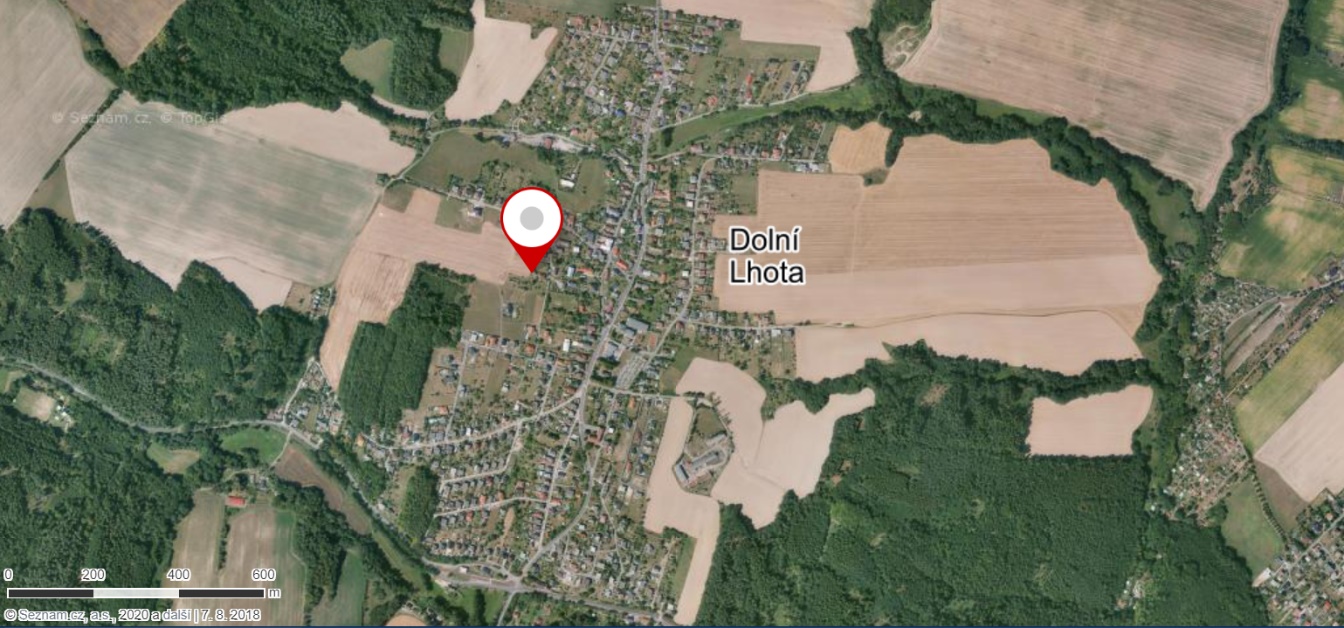  DL [90] |
| 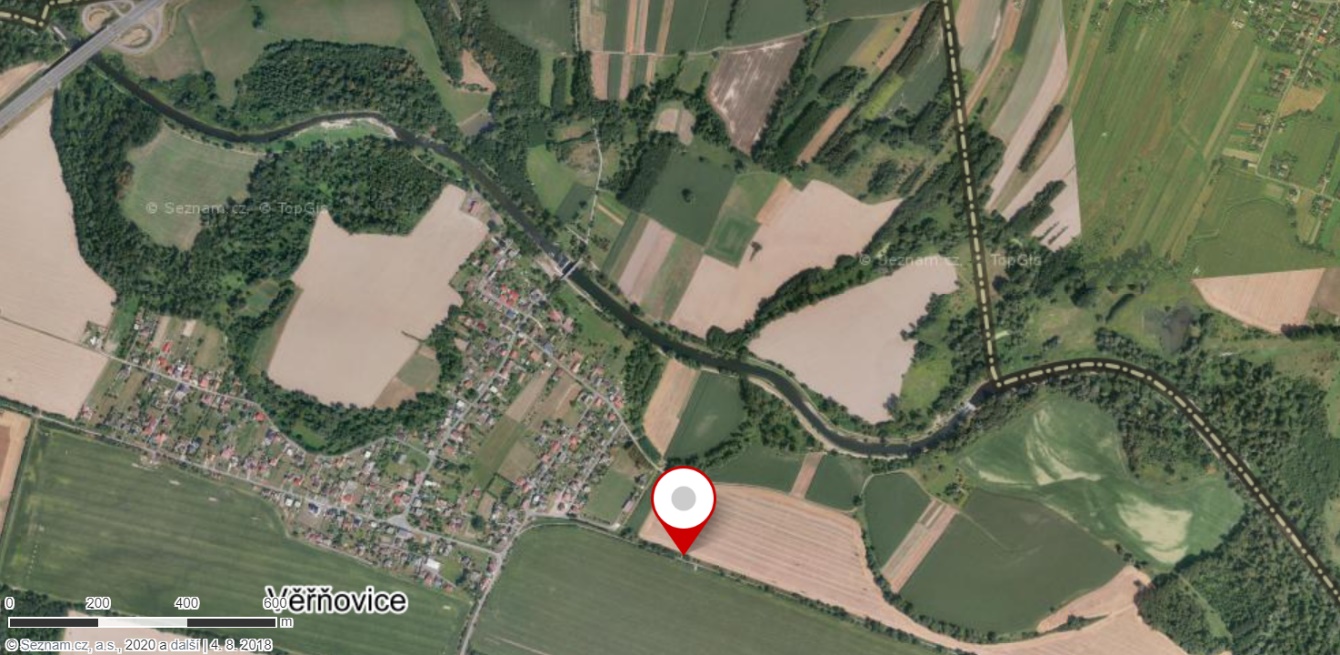  V [90] |
| 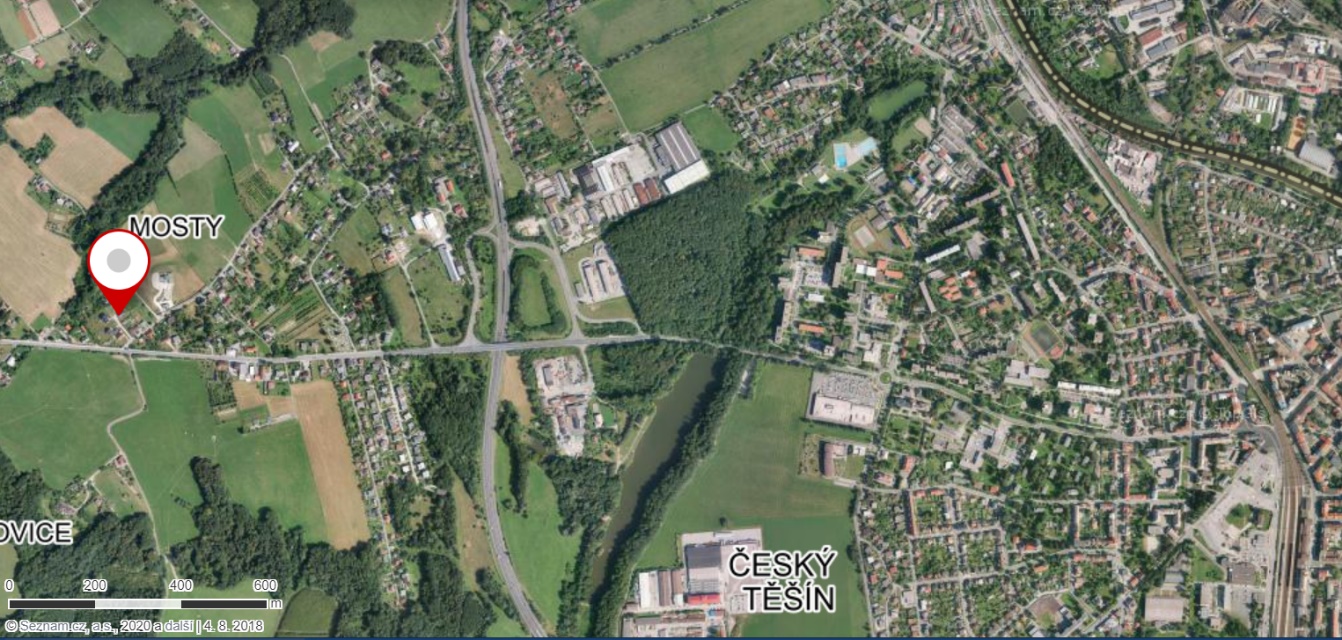  MCT [90] |
| 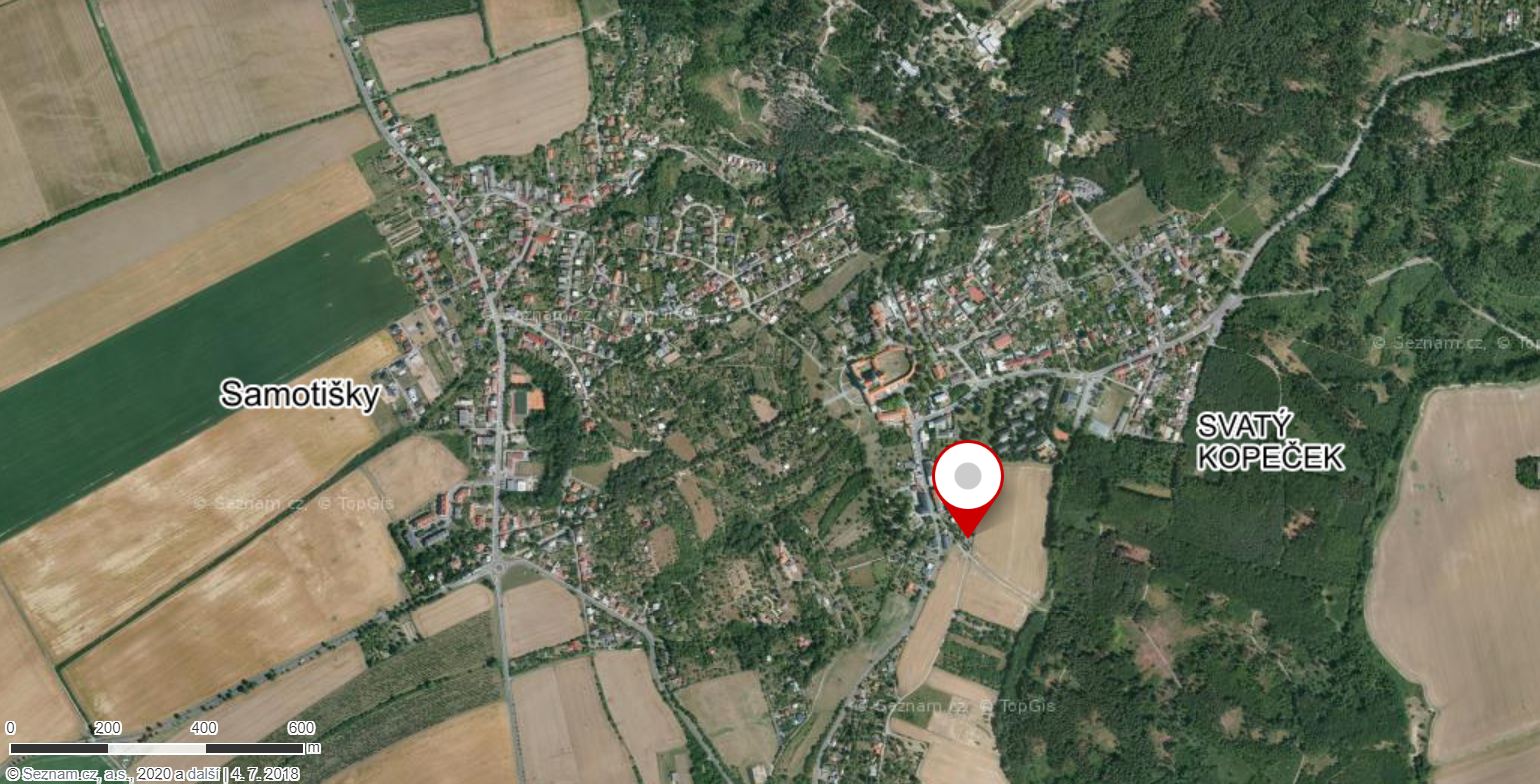  Olomouc [90] |

Source of the figures:

1. Mapy.cz, https://mapy.cz/letecka?x=18.4237417&y=49.8796733&z=10 (Accessed: 20-March-2020).
